# The relationship between autoimmune disorders and intracranial aneurysms in East Asian and European populations: a bidirectional and multivariable two-sample Mendelian randomization study

**DOI:** 10.3389/fneur.2024.1412114

**Published:** 2024-07-12

**Authors:** Chao Tang, Rongcheng Ruan, Bingxiao Pan, Minghong Xu, Jing Huang, Zhaoying Xiong, Zhenxing Zhang

**Affiliations:** ^1^Jinzhou Medical University, Jinzhou, China; ^2^The Second Affiliated Hospital of China Medical University, Shenyang, China; ^3^Department of Nuclear Medicine, Nanchong Central Hospital, Nanchong, China; ^4^Department of Neurosurgery, The First Affiliated Hospital of Jinzhou Medical University, Jinzhou, China

**Keywords:** autoimmune disorders, intracranial aneurysms, Mendelian randomization, SNPs, multivariable Mendelian randomization, causal relationship

## Abstract

**Background:**

It remains unclear about the pathogenesis of intracranial aneurysms (IAs) in the setting of autoimmune disorders (ADs). However, the underlying systemic inflammatory characteristics of ADs may affect IAs through shared inflammatory pathways. Therefore, this study was conducted to explore the relationship between ADs and IAs and assess causal effects.

**Methods:**

In this study, 6 common ADs were included to explore their causal relationship with IAs. Besides, a bidirectional two-sample univariable Mendelian randomization (UVMR) analysis was performed. In addition, the primary analysis was performed by the inverse variance weighted (IVW) and Bayesian weighted Mendelian randomization (BWMR) method, and a series of sensitivity analyses were performed to assess the robustness of the results. Further, the data related to ADs and IAs were collected from open genome-wide association study studies (GWASs) and the Cerebrovascular Disease Knowledge Portal (CDKP) (including 11,084 cases and 311,458 controls), respectively. These analyses were conducted based on both the East Asian and European populations. Moreover, 6 ADs were subject to grouping according to connective tissue disease, inflammatory bowel disease, and thyroid disease. On that basis, a multivariate MR (MVMR1) analysis was further performed to explore the independent causal relationship between each AD and IAs, and an MVMR 2 analysis was conducted to investigate such potential confounders as smoking, alcohol consumption, and systolic blood pressure. Finally, these results were verified based on the data from another GWAS of IAs.

**Results:**

The UVMR analysis results demonstrated that systemic lupus erythematosus (SLE) was associated with a high risk of IAs in the East Asian population (IVW OR, 1.06; 95%CI, 1.02–1.11; *p* = 0.0065, UVMR), which was supported by the results of BWMR (OR, 1.06; 95%CI, 1.02–1.11; *p* = 0.0067, BWMR), MVMR1 (OR, 1.06; 95%CI, 1.01–1.10; *p* = 0.015, MVMR1), MVMR2 (OR, 1.05; 95%CI, 1.00–1.11; *p* = 0.049, MVMR2), and sensitivity analyses. The results in the validation group also suggested a causal relationship between SLE and IAs (IVW OR, 1.04; 95% CI, 1.00–1.09; *p* = 0.046). The reverse MR analysis results did not reveal a causal relationship between IAs and ADs.

**Conclusion:**

In this MR study, SLE was validated to be a risk factor for IAs in the East Asian population. Therefore, the management of IAs in patients with SLE should be highlighted to avoid stroke events.

## 1 Introduction

Intracranial aneurysms (IAs) are defined as locally abnormal enlargement of the lumen of cerebral arteries due to congenital defects or external factors, exhibiting a tumor-like protrusion on the artery wall, namely, cystic or band-like changes in the structure (1, 2). As reported in imaging studies using arteriography and MRI, the incidence of IAs ranges from 0.5 to 3% in the general population. In a prevalence study based on the European population, aneurysms were detected in the MRI-based screening of approximately 1.8% of adult subjects. As per a cross-sectional study in China, 7% of adults aged 35–75 years were diagnosed with aneurysms during brain magnetic resonance angiography screening (3). Although the etiology of IAs has not been defined, their initiation and growth are correlated with flow-related wall shear stress, hereditary factors, and inflammation. It has been suggested that inflammation plays a pivotal role in the occurrence of IAs. Some diseases, such as systemic lupus erythematosus (SLE), rheumatoid arthritis (RA), and Sjögren’s syndrome (SS), known for their systemic inflammatory attributes, might affect the development of IAs through inflammatory pathways (4). Therefore, this study was conducted to explore the relationship between autoimmune disorders (AD) and IAs. These findings may contribute to improving the screening of patients with cerebral aneurysms or identifying new therapeutic targets for the medical management of IAs.

Inflammation may be a major mechanism for impairing the vascular wall and is associated with the development of the aneurysm wall. The formation of abdominal aortic aneurysms has been validated to correlate with such autoimmune diseases as rheumatoid arthritis (RA), hypothyroidism, and SLE (5–7). IAs may follow a similar pathophysiological mechanism. According to several case reports, the causes of cerebral hemorrhage and IAs in people with SLE, RA, multiple sclerosis, sarcoidosis, and Sjögren’s syndrome (SS) may be related to the underlying mechanisms of autoimmune cerebrovascular inflammation (8–13). A smaller size of aneurysms at rupture was found in patients with ADs; Further, patients with ADs had significantly smaller average ruptured aneurysms than those without ADs, according to a retrospective review including 190 patients with ruptured and unruptured cystic IAs (4). As a result, these studies indicate that ADs might wield influence on the development of IAs. However, the impact of diverse confounders (like the usage of steroid hormones in treatment and the presence of hypertension) cannot be eliminated in existing studies. Furthermore, the limited scale of these studies curtails their capacity to thoroughly dissect the causal relationships.

Mendelian randomization (MR) is a causal inference method, which can be employed to adeptly probe the influence of modifiable exposure on diseases. It leverages genetic variation to furnish compelling evidence of robust associations (14). Besides, it harnesses the merits of consolidated statistics originating from extensive genome-wide association studies (GWASs) conducted across sweeping cohorts. Overall, this method makes use of bigger sample sizes and reduces potential biases that may occur in single-sample analyses to increase the accuracy of causal estimations in two-sample MR studies, in which the data from two independent sources are employed. To further clarify the causal relationship between ADs and IAs, multivariate MR (MVMR) and reverse MR analyses were also performed in this study, which may minimize confounders and exclude reverse causality, thus contributing to more stable and reliable results.

## 2 Materials and methods

### 2.1 Study design

**Figure 1:**
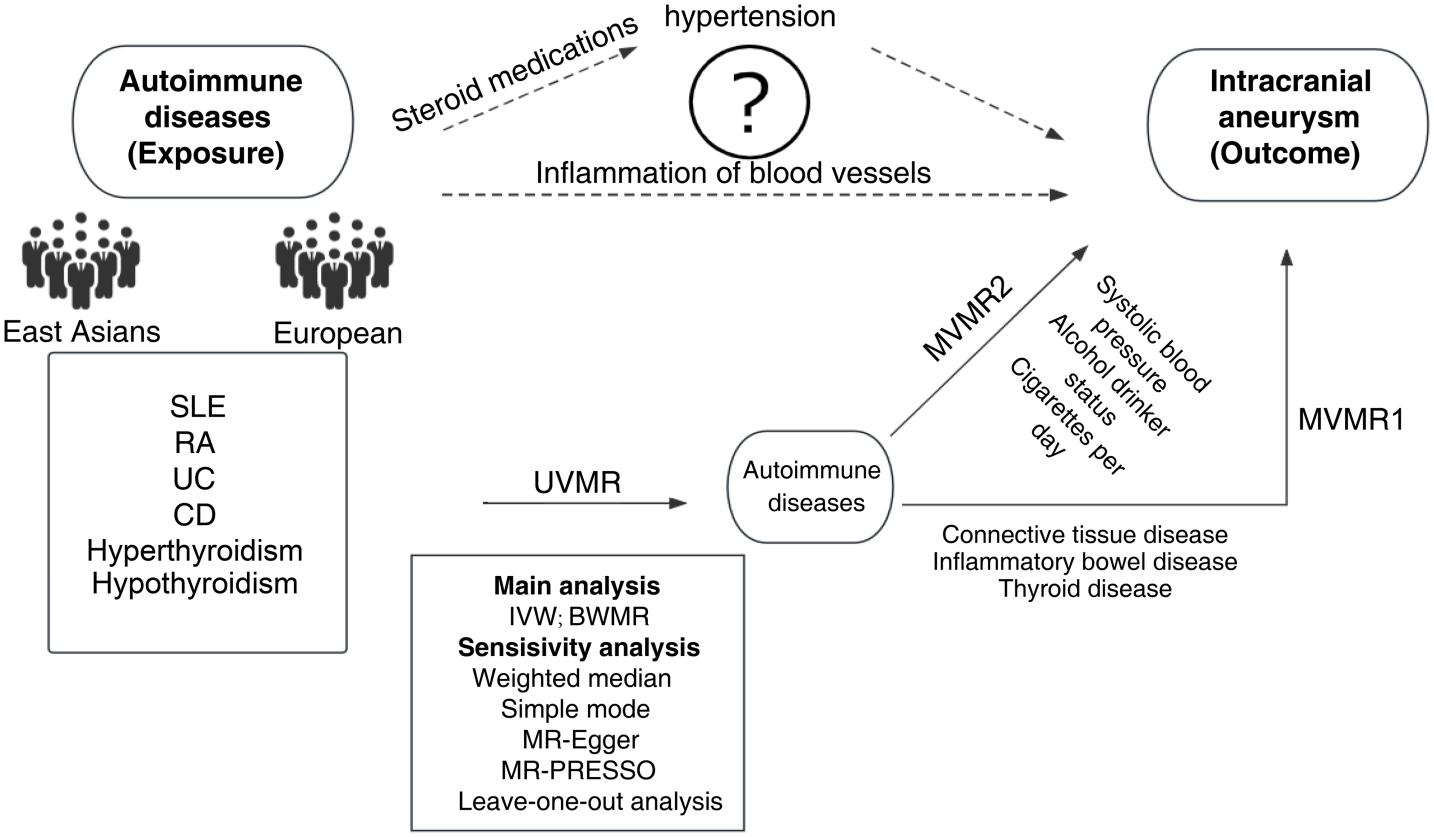
MR design and flowchart of this study. MR, Mendelian randomization; SLE, systemic lupus erythematosus; RA, rheumatoid arthritis; UC, ulcerative colitis; CD, Crohn’s disease; UVMR, univariable Mendelian randomization; MVMR, multivariate Mendelian randomization; BWMR, Bayesian weighted Mendelian randomization; IVW, inverse variance weighted; MR-PRESSO, MR pleiotropy residual sum and outlier test.

Firstly, the instrumental variables (IVs) for ADs and IAs were curated based on summary statistics from both the European and East Asian populations. Besides, UVMR was utilized to estimate the causal impact of ADs on IAs. Additionally, 6 ADs were subject to grouping according to connective tissue disease, inflammatory bowel disease, and thyroid disease. Moreover, MVMR1 was conducted to discern the independent effects of each AD on IAs; MVMR2 was performed on the exposure, including alcohol consumption, smoking, and systolic blood pressure, to avoid the influence of confounders (15). To further fortify our analysis, a reverse MR was conducted to counteract the potential effects of reverse causality. The study design process is illustrated in Figure 1. Furthermore, another GWAS of IAs was selected for validation. The MR analysis should be performed based on three core assumptions: (1) there is a strong correlation between IVs and exposure; (2) there is independence between IVs and confounders; and (3) IVs can only exert an effect on outcomes through exposure.

### 2.2 GWAS summary data for ADs

In this study, 6 common ADs, including SLE, RA, ulcerative colitis (UC), Crohn’s disease (CD), hyperthyroidism, and hypothyroidism, were included to explore the relationship between ADs and IAs. These data can be obtained from the website^1^ based on the GWAS ID in Table 1 or in the original GAWS article (16–19), which contained the GWAS data that were available to the public and did not require an ethical review. To acquire more significant research and dependable results, the data with the biggest sample size were also selected and then stratified based on the races in the European and East Asian populations. The details of these data are summarized in Table 1.

**Table 1:** Characteristics of data in this study.

Trait		Sample size (case and controls)	Population	GWAS ID (PMID)
Autoimmune disorders	SLE	14,267 (5,201 and 9,066)	European	ebi-a-GCST003156 (26502338)
	RA	417,256 (8,255 and 409,001)		ebi-a-GCST90018910 (34594039)
	UC	417,932 (5,371 and 412,561)		ebi-a-GCST90018933 (34594039)
	CD	20,883 (5,956 and 14,927)		ieu-a-30 (26192919)
	Hyperthyroidism	460,499 (3,557 and 456,942)		ebi-a-GCST90018860 (34594039)
	Hypothyroidism	410,141 (30,155 and 379,986)		ebi-a-GCST90018862 (34594039)
Autoimmune disorders	SLE	12,653 (4,222 and 8,431)	East Asian	ebi-a-GCST90011866 (33536424)
	RA	19,190 (3,636 and 15,554)		bbj-a-72 (24390342)
	UC	4,853 (1,134 and 3,719)		ieu-a-969 (26192919)
	CD	5,409 (1,690 and 3,719)		ieu-a-11 (26192919)
	Hyperthyroidism	173,650 (994 and 172,656)		ebi-a-GCST90018640 (34594039)
	Hypothyroidism	173,770 (1,114 and 172,656)		ebi-a-GCST90018642 (34594039)
Intracranial Aneurysm	uIA and IA	317,636 (11,084 and 311,458)	Mixed	https://cd.hugeamp.org/datasets.html (33199917)
		79,429 (6,242 and 63,994)	European	
		238,207 (3,259 and 234,946)	East Asian	
	IA (validation groups)	473,683 (945 and 472,738)	European	ebi-a-GCST90018815
		195,203 (2,820 and 192,383)	East Asian	bbj-a-96
Risk factors	Cigarettes per day	4,772	European	ebi-a-GCST009966 (32157176)
	Alcohol drinker status	360,726 (336,919 and 23,807)		ukb-d-20117_2
	Systolic blood pressure	422,713		ebi-a-GCST90025968 (34226706)
	cigarettes per day	72,655	East Asian	ieu-b-5071 (31089300)
	Alcohol drinker status: Current	2,656 (555 and 2,101)		ukb-e-20117_p1_EAS
	Systolic blood pressure	145,505		ieu-b-5075 (34594039)

SLE, systemic lupus erythematosus; RA, rheumatoid arthritis; UC, ulcerative colitis; CD, Crohn’s disease; IA, intracranial aneurysm; uIA, unruptured intracranial aneurysm.

### 2.3 GWAS summary data for IAs

A GWAS including 11,084 cases and 311,458 controls of the European and East Asian populations yielded summary statistics for IAs. The summary data were collected from the CDKP (20). Among the GWAS summary data of IAs (ruptured and unruptured) (*n* = 6,242) against controls (*n* = 63,994) in the European population and those of IAs (ruptured and unruptured) (*n* = 3,259) against controls (*n* = 234,946) in the East Asian population, there were 2,662 cases and 164,009 controls from Biobank Japan (BBJ) and 597 cases and 70,939 controls from China Kadoorie Biobank (CKB).

In the validation group, the GWAS data of IAs were obtained from the OpenGWAS database, including 945 cases and 472,738 controls in the European population and 2,820 cases and 192,383 controls in the East Asian population. All details of these data are summarized in Table 1.

### 2.4 IV selection

Single nucleotide polymorphisms (SNPs), as IVs with a good correlation with each AD, were used in this MR study. Besides, a variety of quality control strategies were employed to filter eligible genetic IVs that conformed to the three main MR assumptions. Firstly, the SNPs associated with ADs were isolated from the entire genome (*p* < 5 × 10^−8^). For hyperthyroidism and hypothyroidism in the East Asian population, a threshold of *p* < 5 × 10^−6^ was set for more IVs. In addition, a threshold of r^2^ = 0.001 was used to reduce linkage imbalance and trimmed SNPs within a window size of 10,000 kb to ensure the independence of each IV (LD). Then, the pool of IVs and SNPs that were missing from the results was purged of palindromic SNPs. The same criteria were adopted in the validation group. Lastly, the F statistic for IVs was calculated to identify whether there was weak instrumental bias. Using the formula F = β^2^_exposure_/SE^2^_exposure_ (21), the F statistic was larger than 10, proving that there was no bias due to weak IVs (22).

### 2.5 Statistical analysis

R software 4.3.1, *BWMR*, and *TwoSampleMR* were used in this study. The IVW approach is the primary focus of UVMR, and it can be applied in the absence of potential horizontal pleiotropism (23). BWMR is causally inferred by the variational expectation–maximization (VEM) algorithm, which further considers the uncertainty of weak effects. The pleiotropy has been addressed by BWMR outlier detection, and the BWMR results are also reliable (24). In this study, MVMR derived from UVMR was also performed. In regression models, some key variables and outcomes are often used to perform univariate regression first, followed by the addition of confounders to conduct multivariate regression for corrections. The same is true in MR, which can be used to correct confounders, especially when multiple SNPs overlap. Subsequently, MR-Egger, simple mode, weighted median, and weighted model techniques were added to the IVW results (25). As long as the beta values of other methods are aligned in the same direction, significant results from the IVW method are taken into consideration meaningfully even when other methods are not. After the Bonferroni correction, a *p* value below 0.008 (0.05/6) was considered statistically significant. The presence of horizontal pleiotropy was identified using MR-Egger intercepts (26). Level pleiotropy outliers may be found using the MR-PRESSO framework, and outlier removal can be utilized to correct IVW estimates (27). To verify whether a particular outlier variable had an impact on the effect estimates, a stay-aside analysis was carried out. Moreover, Cochran’s Q test was performed by IVW and MR-Egger analyses to assess the heterogeneity of each SNP, and heterogeneity was indicated by Q-statistics with a *p*-value <0.05.

## 3 Results

### 3.1 MR results in the European population

**Figure 2:**
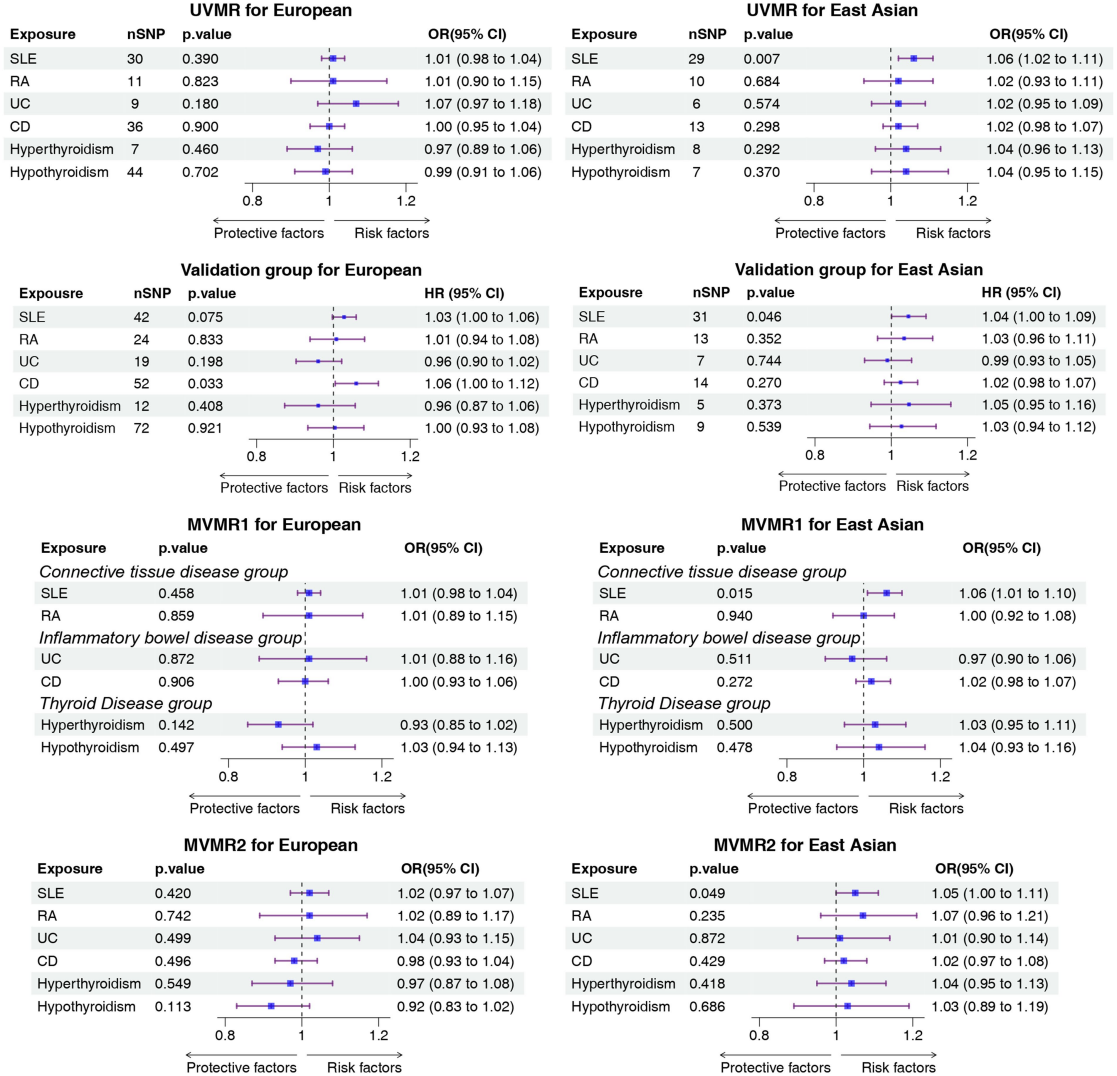
Forest plot of the causal association between ADs and IA. ADs, autoimmune disorders; IA, intracranial aneurysms; UVMR, univariable mendelian randomization; MVMR, multivariable mendelian randomization; OR, odds ratio; SLE, systemic lupus erythematosus; RA, rheumatoid arthritis; UC, ulcerative colitis; CD, Crohn’s disease.

In this study, 30, 11, 9, 36, 7, and 44 SNPs were incorporated in the UVMR for SLE, RA, UC, CD, hyperthyroidism, and hypothyroidism, respectively, within the European population. The F-statistic values of these SNPs all exceeded 10, with the average F-statistic values being 86.43, 52.10, 44.93, 64.97, 58.16, and 80.13, respectively, as listed in Supplementary Table S1. The absence of weak IVs was evident. However, no causal relationship between ADs and IAs was identified in the European population, as indicated by *p*-values exceeding 0.05 in each case (Figure 2; Supplementary Tables S2–S4). The scatter plot and forest plot of each outcome are illustrated in Supplementary Figures S2, S3. The MVMR results also revealed no causal relationship between ADs and IAs (Figure 2). The results of all BWMR analyses are presented in Supplementary Figures S4–S9. Similarly, the reverse MR analysis failed to reveal a causal relationship between IAs and ADs (Supplementary Tables S6, S7). Moreover, multiple sensitivity analyses were also conducted, revealing horizontal pleiotropy in hypothyroidism and IAs results, heterogeneity in CD and IAs results, and outliers identified by the MR-PRESSO analysis (Supplementary Table S5). In the reverse MR analysis, the MR-Egger method indicated no horizontal pleiotropy; the Q-test demonstrated heterogeneity in the results of SLE, UC, and CD; MR-PRESSO highlighted outliers in the results of UC and CD (Supplementary Table S8). The leave-one-out analysis confirmed that individual SNPs did not drive the results, as depicted in Supplementary Figure S1.

### 3.2 MR results in the East Asian population

In this study, 29, 10, 6, 13, 8, and 7 SNPs were incorporated for SLE, RA, UC, CD, hyperthyroidism, and hypothyroidism, respectively, in the East Asian population. The F-statistic values of these SNPs all exceeded 10, with the average F-statistic values being 63.07, 67.77, 61.71, 72.63, 33.61, and 23.19, respectively (Supplementary Table S1). This confirmed the absence of weak IVs. According to the UVMR results, a statistically significant relationship between IA incidence and SLE was identified in the East Asian population (IVW OR, 1.06; 95% CI, 1.02–1.11; *p* = 0.0065, UVMR). This result was supported in BWMR (OR, 1.06; 95%CI, 1.02–1.11; *p* = 0.0067, BWMR) and MVMR1 (IVW OR, 1.06; 95% CI, 1.01–1.10; *p* = 0.015, MVMR1). In MVMR2, the relationship between SLE and IAs was weak but remained statistically significant (IVW OR, 1.05; 95% CI, 1.00–1.11; *p* = 0.049, MVMR2) (Figure 2; Supplementary Tables S2–S4). The scatter plot and forest plot of each outcome are depicted in Supplementary Figures S2, S3. The results of all BWMR analyses are presented in Supplementary Figures S10–S15. The reverse MR analysis did not reveal a causal relationship between IAs and ADs (Supplementary Tables S6, S7). Moreover, sensitivity analyses were also conducted, revealing no heterogeneity and no horizontal pleiotropy in all results, which was also supported by the MR-PRESSO results (Supplementary Tables S5, S8). Furthermore, it was also confirmed that the causal effect was not driven by a single SNP (Supplementary Figure S2).

### 3.3 Validation groups

In the East Asian population, the MR results suggested a causal relationship between SLE and IAs (IVW OR, 1.04; 95% CI, 1.00–1.09; *p* = 0.046), and this result was also supported by sensitivity analyses. In the European population, the MR results suggested a causal relationship between CD and IAs, but the MR-PRESSO results indicated the presence of pleiotropy. Relevant results are summarized in Figure 2, and detailed results are listed in the Supplementary materials.

## 4 Discussion

To the best of our knowledge, this is the first study to employ the MR method and GWAS summary statistics to investigate the causal relationship between ADs and the risk of intracranial aneurysms (IAs) in both the European and East Asian populations. Within the univariable Mendelian randomization (UVMR) and MVMR analyses, a heightened incidence of IAs was observed in systemic lupus erythematosus (SLE) within the East Asian population. This result was also supported in the validation group. However, there was no causal relationship identified between the other five ADs and IAs in both populations.

Currently, it remains unclear about the pathogenesis of IAs, particularly in the context of ADs. It has been validated in numerous epidemiological studies on IAs that IAs have a potential link with the occurrence and development of ADs (3). In the absence of pathological confirmation, most studies have proposed two hypotheses to explain the reason why ADs increase the risk of IAs (4, 28, 29) (Figure 1). IAs may result from inflammatory pathways, which may be potentially exacerbated by ADs. Hemodynamic stress can cause endothelial damage, and endothelial dysfunction along with vasculitis may contribute to the development of IAs (30, 31). The infiltration of inflammatory cells, including macrophages, monocytes, mast cells (32), and T lymphocytes, as well as complement activation (32–38), has been confirmed in all IAs. Berry aneurysms, or parts thereof, are reported to be autoimmune and can be attributed to inflammatory diseases in the blood vessels. This is partially supported by the identification of inflammatory infiltrates in the wall of IAs (39). A case–control study revealed an independent relationship between hypothyroidism and unruptured intracranial aneurysm (uIA). Autoimmune hypothyroidism may induce endothelial dysfunction through a chronic inflammatory mechanism mediated by inflammatory cytokines (6). IAs may be linked to the administration of steroid drugs in patients with ADs. Classic risk factors such as atherosclerosis and hypertension are complications associated with SLE patients (29, 40). Steroids and/or cytotoxic immunosuppressants are used in the treatment of SLE in the acute phase. The side effects of these agents can also lead to hypertension. Saccular aneurysms are associated with atherosclerosis and hypertension. In a Taiwanese study, the administration of relatively high average daily doses of steroids was identified as an independent risk factor for the increased risk of subarachnoid hemorrhage (SAH) in SLE patients (41). These two hypotheses are purely theoretical speculations. They may be independent or they may represent common pathways leading to the occurrence of IAs. Combined with the findings of this study, it is necessary to conduct further experimental verification.

As an autoimmune disease, SLE is more common in young females and exhibits the highest incidence in China (about 0.07%) (42). Numerous studies have found an association between SLE and IAs (29, 41, 43, 44). In a study comparing the cohorts of immune-mediated diseases with the control cohorts to calculate the SAH rate, patients with SLE exhibited the highest risk among all immune-mediated diseases (RR = 3.76, 3.08–4.55) (28). Endothelial dysfunction and vasculitis in SLE patients may contribute to the development of IAs (45, 46). In contrast to the general population, the proportion of saccular aneurysms is lower in SLE patients, while more common aneurysms such as spindle aneurysms or pseudoaneurysms are prevalent among these patients (29, 46, 47). Typically, patients experiencing SAH with vascular-negative aneurysms tend to be older and have a benign clinical course (48). Conversely, SLE patients presenting with SAH and angionegative aneurysms are usually relatively young and face a high mortality rate (29, 46, 47). The higher incidence of multiple aneurysms is another characteristic of SLE patients, with the incidence of single and multiple aneurysms in SLE patients being 69.5 and 31.6%, respectively (49).

The mechanisms by which SLE leads to IAs have not been defined. Most scholars attribute IAs to the inflammation of intracranial arteries or artery walls caused by SLE, but the specific molecular mechanisms have not been sufficiently clarified, which requires further basic experimental research. In large-scale studies on SLE in North America and Europe, the incidence of clinically defined SAH in patients with central nervous system involvement is about 0.3% (50–53). A Japanese study showed that the prevalence of SLE in the Japanese population and the prevalence of SHA in SLE patients were 1.28 and 3.9%, respectively, which were higher than those in Western countries (53). In this study, a correlation between SLE and IAs was only found in the East Asian population. However, the reasons and mechanisms for this difference between the two populations are currently unknown. It may be attributed to the differences in the data itself or inherent racial differences, which should be explored in further research.

In contrast to previous observational studies, several critical advantages augment the credibility and robustness of the conclusions in this study. Firstly, to the best of our knowledge, this study represents the first attempt to explore the causal relationship between ADs and IAs through a two-sample MR analysis. This innovative approach enabled us to thoroughly and rigorously examine the causal effects of ADs on IAs. Additionally, a range of sophisticated MR techniques, encompassing bidirectional MR analysis, MVMR analysis, and MR-PRESSP methods, were used in this study. The bidirectional MR analysis allowed the scrutinization of the potential influence of reverse causality, while MVMR further enabled an exploration of intricate interactions between ADs and IAs. The MR-PRESSO method effectively avoided potential abnormalities. Furthermore, a series of stringent sensitivity analyses, including checks for heterogeneity and pleiotropy, were conducted to substantiate the reliability of our results. Finally, our results were also validated in the validation group to make the results more reliable.

Nevertheless, there are still several inherent limitations in this study. Firstly, we conducted relevant explorations based on the East Asian and European populations, not global populations, and hence caution is needed when applying the results to other populations. Secondly, the results of our study differed in both populations. Therefore, further research is needed to identify the significance of this difference. Thirdly, the aneurysm GWAS data including both uIA and ruptured IAs were collected in this study, but data limitations cannot be avoided. Fourthly, although causal effects can be derived by MR methods, the limitations of this study are still obvious due to a lack of prospective studies. Finally, only 6 ADs were included in this study, and there may be other ADs associated with IAs, which may be attributed to a lack of data on autoimmune disease-associated GWASs in the East Asian population. After the data that were pleiotropic and unreliable in results were excluded, these 6 autoimmune diseases were incorporated into this study, thus ensuring the consistency in the studies in both populations.

## 5 Conclusion

In conclusion, the results of this study suggested that SLE was associated with a higher risk of IAs in the East Asian population. This underscored the pivotal clinical importance for healthcare practitioners to promptly recognize and diagnose IA patients within the framework of SLE. Therefore, strengthening cerebrovascular disease management in individuals with SLE becomes imperative, which may contribute to mitigating the morbidity and mortality associated with aneurysmal SAH in this patient population.

## Data availability statement

The datasets presented in this study can be found in online repositories. The names of the repository/repositories and accession number(s) can be found in the article/Supplementary material.

## Ethics statement

Ethical review and approval was not required for the study on human participants in accordance with the local legislation and institutional requirements. Written informed consent from the patients/participants or patients/participants' legal guardian/next of kin was not required to participate in this study in accordance with the national legislation and the institutional requirements.

## Author contributions

CT: Conceptualization, Data curation, Software, Visualization, Writing – original draft, Writing – review & editing. RR: Methodology, Writing – original draft. BP: Conceptualization, Investigation, Writing – original draft. MX: Project administration, Validation, Writing – original draft. JH: Data curation, Resources, Writing – review & editing. ZX: Formal analysis, Supervision, Writing – original draft. ZZ: Data curation, Funding acquisition, Supervision, Validation, Writing – review & editing.

## Footnotes

^1^ https://gwas.mrcieu.ac.uk/
