# Supplementary material for: The relationship between autoimmune disorders and intracranial aneurysms in East Asian and European populations: a bidirectional and multivariable two-sample Mendelian randomization study
Source: Front Neurol. 2024 Jul 12;15:1412114. doi: 10.3389/fneur.2024.1412114 (PMC11272522; doi:10.3389/fneur.2024.1412114)
Supplement: Supplementary file 1 [file Data_Sheet_1.ZIP › Supplementary Material/Source code.docx]

#UVMR:

library(TwoSampleMR)

library(data.table)

sle_exp_dat<- extract_instruments(outcomes = 'ebi-a-GCST90011866',

p1 = 5e-08,

clump = TRUE, r2 = 0.001,

kb = 10000, access_token = NULL)

library(data.table)

outcome<-fread('IA.GWAS.BakkerMK.2020.sumstats.EastAsianSubset.txt.gz',header=T)

IA<-format_data(sle_exp_dat,type = "outcome",

header = TRUE,

snps =sle_exp_dat$SNP,

snp_col = "SNP",

beta_col = "BETA",

se_col = "SE",

effect_allele_col = "A_EFF",

other_allele_col = "A_NONEFF",

pval_col = "P")

dat<-harmonise_data(

exposure_dat=sle_exp_dat,

outcome_dat=IA,

action= 2)

res <- mr(dat)

OR <-generate_odds_ratios(res)

het <- mr_heterogeneity(dat)

pleio <- mr_pleiotropy_test(dat)

library(MRPRESSO)

mr_presso(BetaOutcome ="beta.outcome", BetaExposure = "beta.exposure", SdOutcome ="se.outcome", SdExposure = "se.exposure", OUTLIERtest = TRUE,DISTORTIONtest = TRUE, data = dat, NbDistribution = 1000, SignifThreshold = 0.05)

single <- mr_leaveoneout(dat)

mr_leaveoneout_plot(single)

mr_scatter_plot(res,dat)

res_single <- mr_singlesnp(dat)

mr_forest_plot(res_single)

F<-dat$beta.exposure^2/dat$se.exposure^2

write.csv(dat, "C:sle dat.csv")

write.csv(OR, "C:sle OR.csv")

write.csv(het, "C:sle het.csv")

write.csv(pleio, "C:sle pleio.csv")

#BWMR:

library(ggplot2)

source("BWMR_updated.R")

library(BWMR)

a<-read.csv(file= "sle dat.csv",header=T)

myBWMR<- BWMR(gammahat = a$beta.exposure,

Gammahat = a$beta.outcome,

sigmaX = a$se.exposure,

sigmaY = a$se.outcome)

myBWMR

beta=myBWMR[["beta"]]

lci95<-myBWMR[['beta']]-1.96*myBWMR[["se_beta"]]

uci95<-myBWMR[['beta']]+1.96*myBWMR[["se_beta"]]

or <-exp(myBWMR[["beta"]])

or_lci95<-exp(lci95)

or_uci95<-exp(uci95)

pval<-myBWMR[["P_value"]]

se=myBWMR[["se_beta"]]

myres=data.frame(methed="BWMR",beta,se,pval,lci95,uci95,or,or_lci95,or_uci95)

write.csv(myres, "C:sle BWMR.csv")

#MVMR1:

id_exposure<-c("ebi-a-GCST90011866","bbj-a-72")

exposure_dat <- mv_extract_exposures(id_exposure, pval_threshold = 5e-8)

IA1 <-fread('IA.GWAS.BakkerMK.2020.sumstats.EastAsianSubset.txt.gz',header=T)

f<-format_data(IA1,type = "outcome",

header = TRUE,

snps = exposure_dat$SNP,

snp_col = "SNP",

beta_col = "BETA",

se_col = "SE",

effect_allele_col = "A_EFF",

other_allele_col = "A_NONEFF",

pval_col = "P",

log_pval = FALSE)

mvdat <- mv_harmonise_data(exposure_dat,f)

res <- mv_multiple(mvdat)

res_OR<-generate_odds_ratios(res$result)

write.csv(res_OR, "C:1SLE OR.csv")

#MVMR2:

id_exposure2<-c("ebi-a-GCST90011866","ieu-b-5071","ukb-e-20117_p1_EAS","ieu-b-5075")

exposure_dat <- mv_extract_exposures(id_exposure, pval_threshold = 5e-8)

IA1 <-fread('IA.GWAS.BakkerMK.2020.sumstats.EastAsianSubset.txt.gz',header=T)

f1<-format_data(IA1,type = "outcome",

header = TRUE,

snps = exposure_dat$SNP,

snp_col = "SNP",

beta_col = "BETA",

se_col = "SE",

effect_allele_col = "A_EFF",

other_allele_col = "A_NONEFF",

pval_col = "P",

log_pval = FALSE)

mvdat <- mv_harmonise_data(exposure_dat,f1)

res <- mv_multiple(mvdat)

res_OR<-generate_odds_ratios(res$result)

write.csv(res_OR, "C:2SLE OR.csv")

Reverse MR:

library(data.table)

exposure_ia<-fread('IA.GWAS.BakkerMK.2020.sumstats.EastAsianSubset.txt.gz',header=T)

b<-format_data(exposure_ia,type = "exposure",

header = TRUE,

snp_col = "SNP",

beta_col = "BETA",

se_col = "SE",

effect_allele_col = "A_EFF",

other_allele_col = "A_NONEFF",

pval_col = "P",)

c<-subset(b,pval.exposure<5e-6)

d<-clump_data(c,clump_r2=0.001,clump_kb=10000)

e <- extract_outcome_data(d$SNP, outcomes="ebi-a-GCST90011866")

dat <- harmonise_data(

exposure_dat=d,

outcome_dat=e,

action= 2

)

res <- mr(dat)

OR <-generate_odds_ratios(res)
