# Supplementary material for: The relationship between autoimmune disorders and intracranial aneurysms in East Asian and European populations: a bidirectional and multivariable two-sample Mendelian randomization study
Source: Front Neurol. 2024 Jul 12;15:1412114. doi: 10.3389/fneur.2024.1412114 (PMC11272522; doi:10.3389/fneur.2024.1412114)
Supplement: Supplementary file 1 [file Data_Sheet_1.ZIP › Supplementary Material/Supplementary Figure.docx]

1a
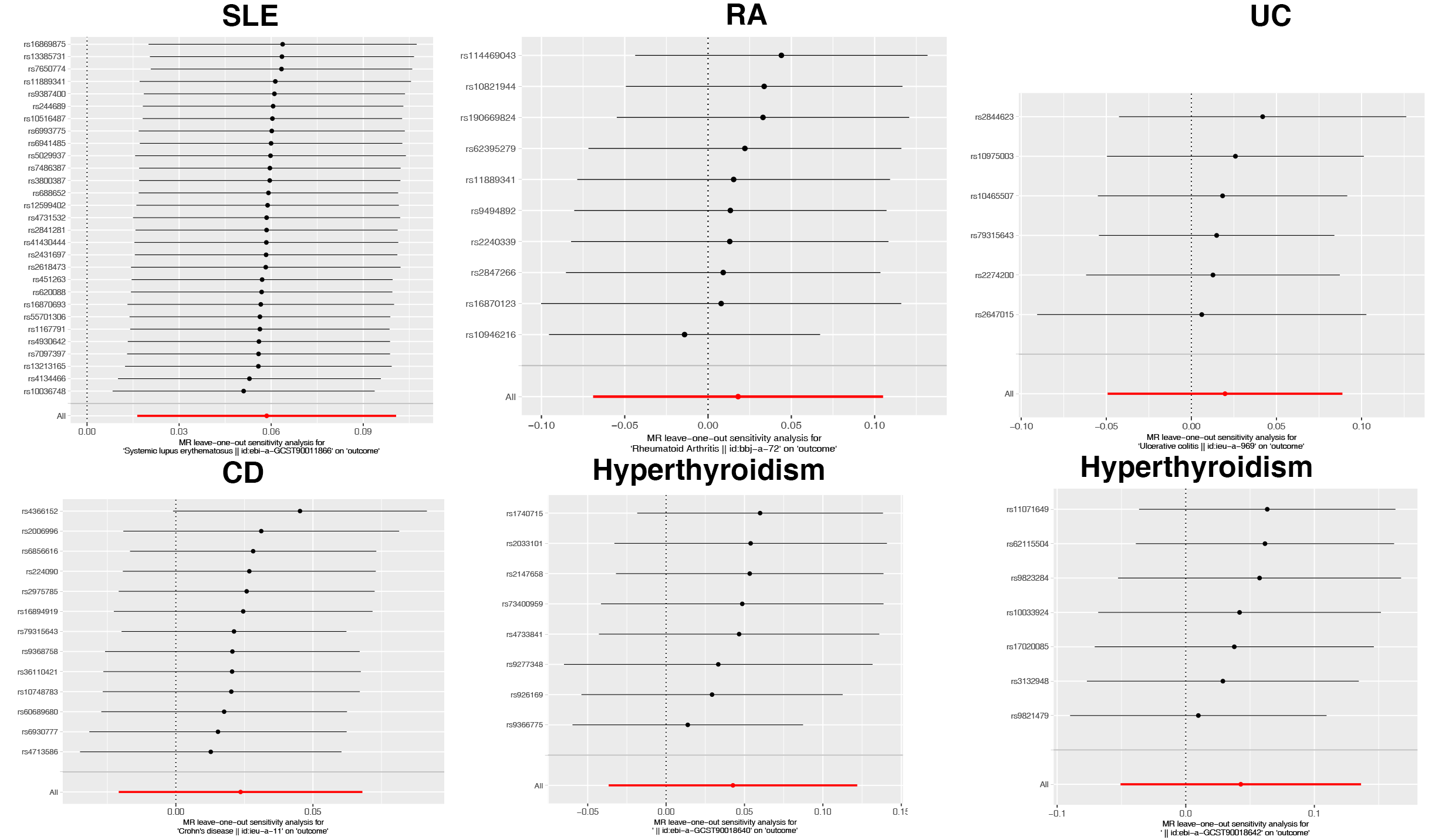


**Supplementary Figure 1a：Leave-one-out test plot of causal effect estimates for European.**

1b
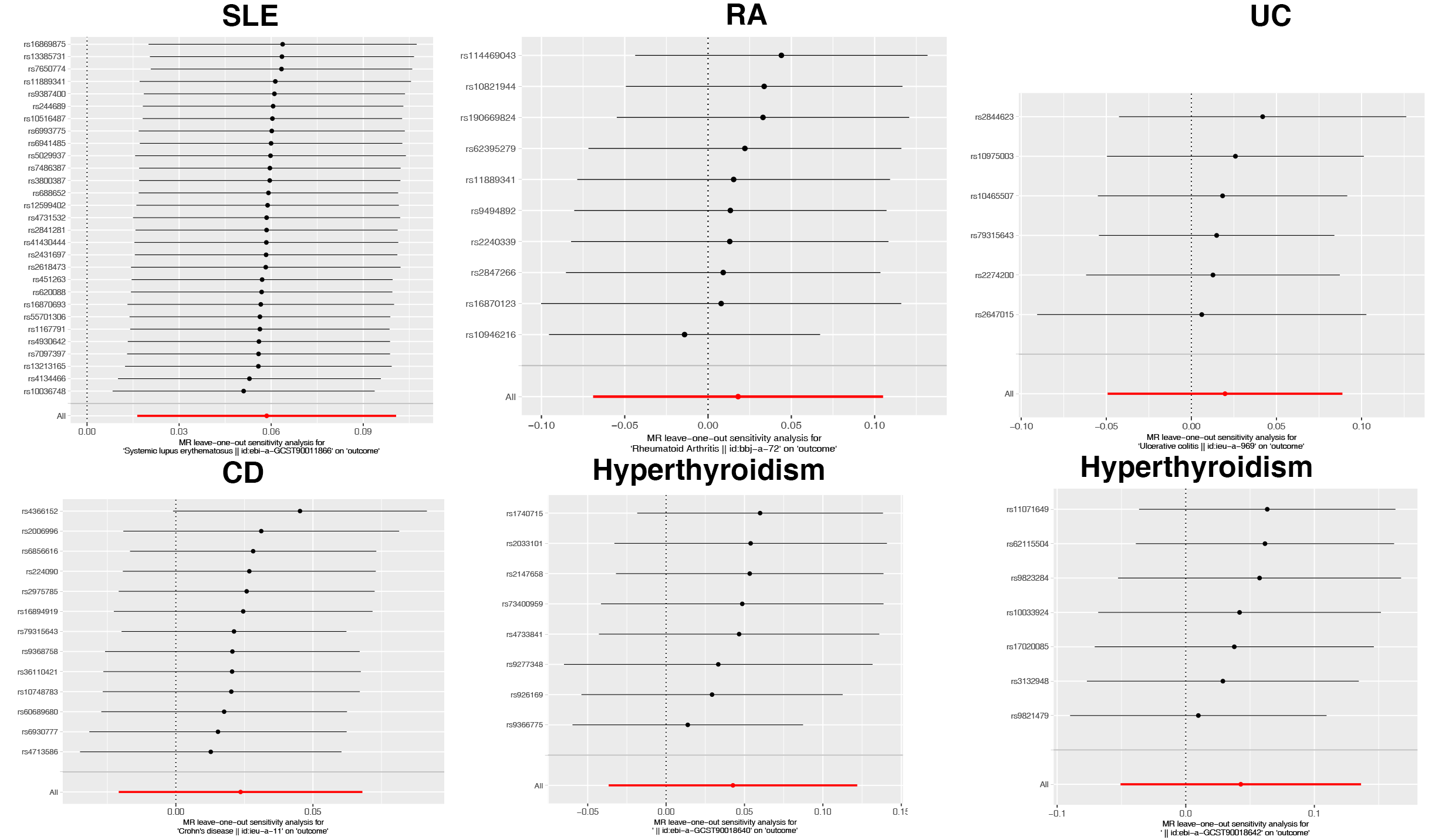


**Supplementary Figure 1b：Leave-one-out test plot of causal effect estimates for East Asian.**

1c
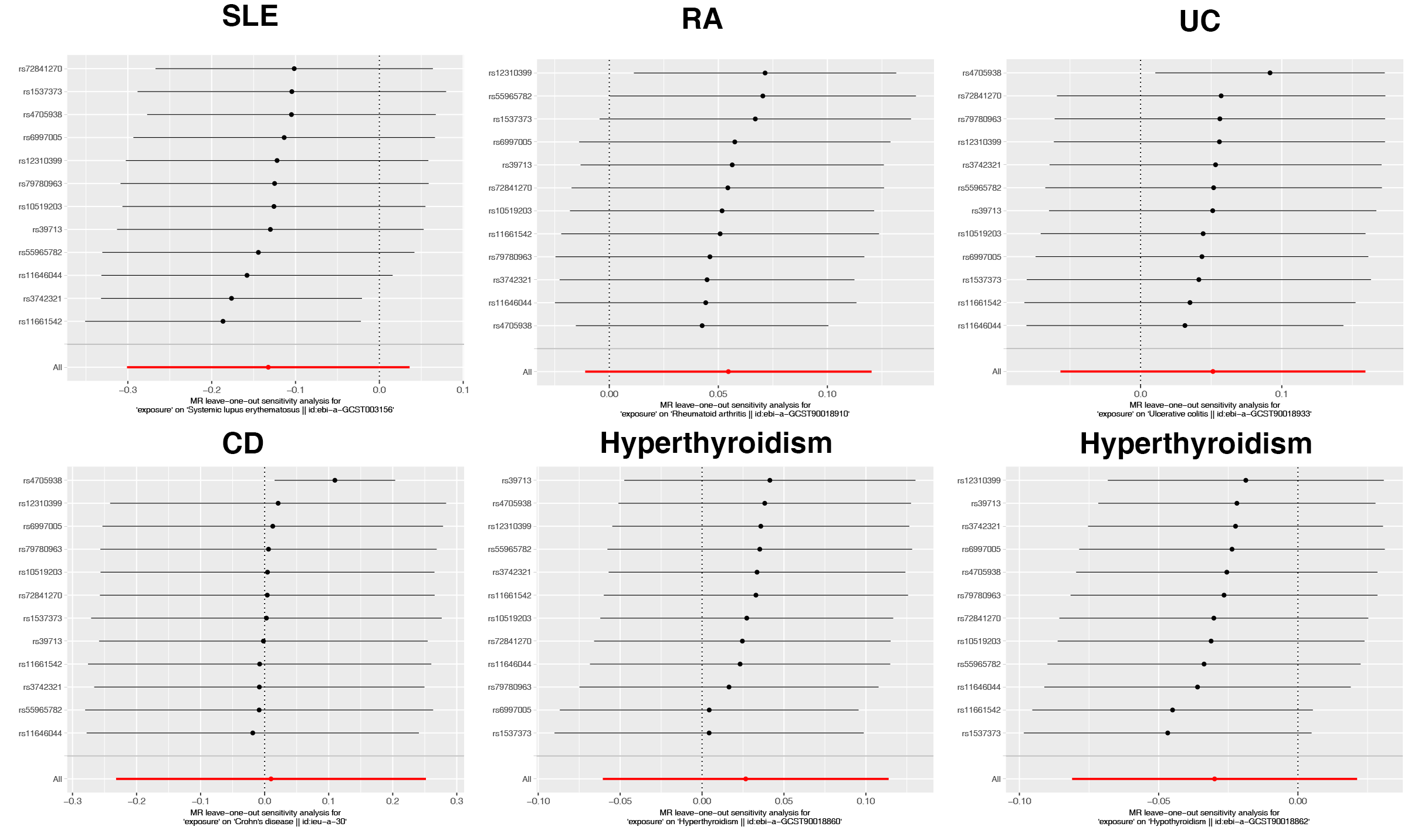


**Supplementary Figure 1c：Leave-one-out test plot of causal effect estimates for European in reverse MR.**

1d
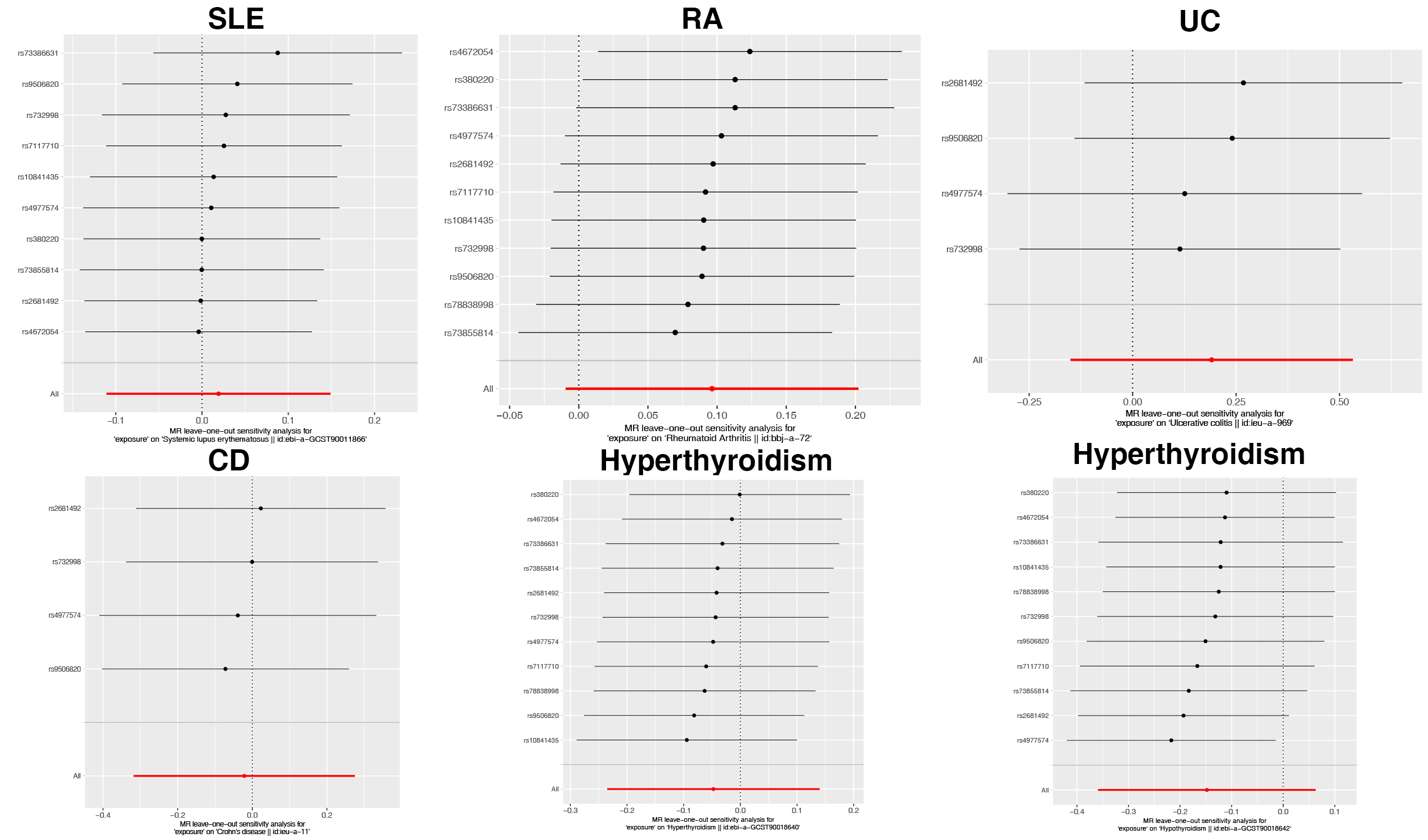


**Supplementary Figure 1d：Leave-one-out test plot of causal effect estimates for East Asian in reverse MR.**

SLE, Systemic lupus erythematosus; RA, Rheumatoid arthritis; UC, Ulcerative colitis; CD, Crohn's disease;

2a


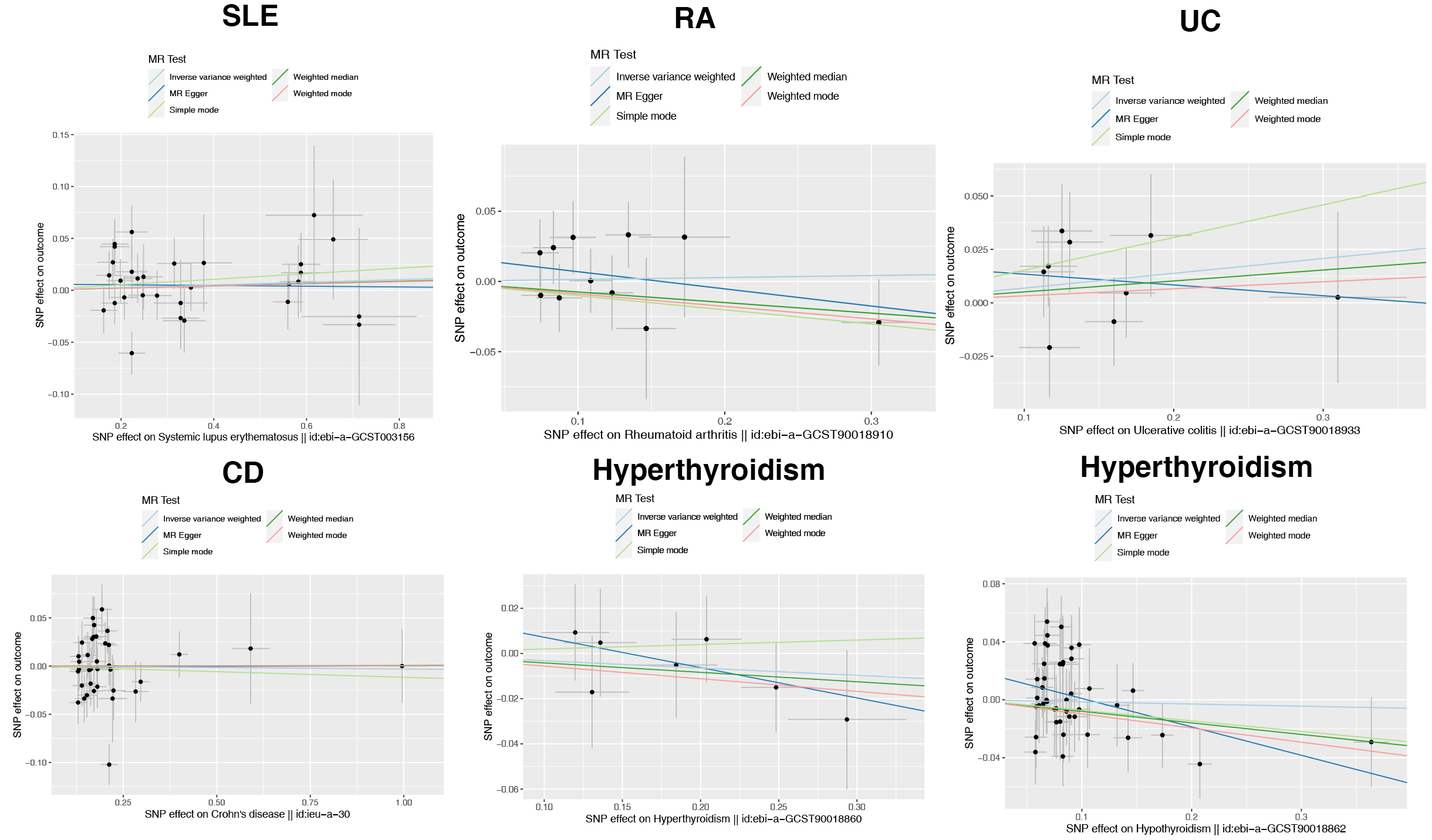


**Supplementary Figure 2a:Scatter plots of causal effect estimates for European.**

2b


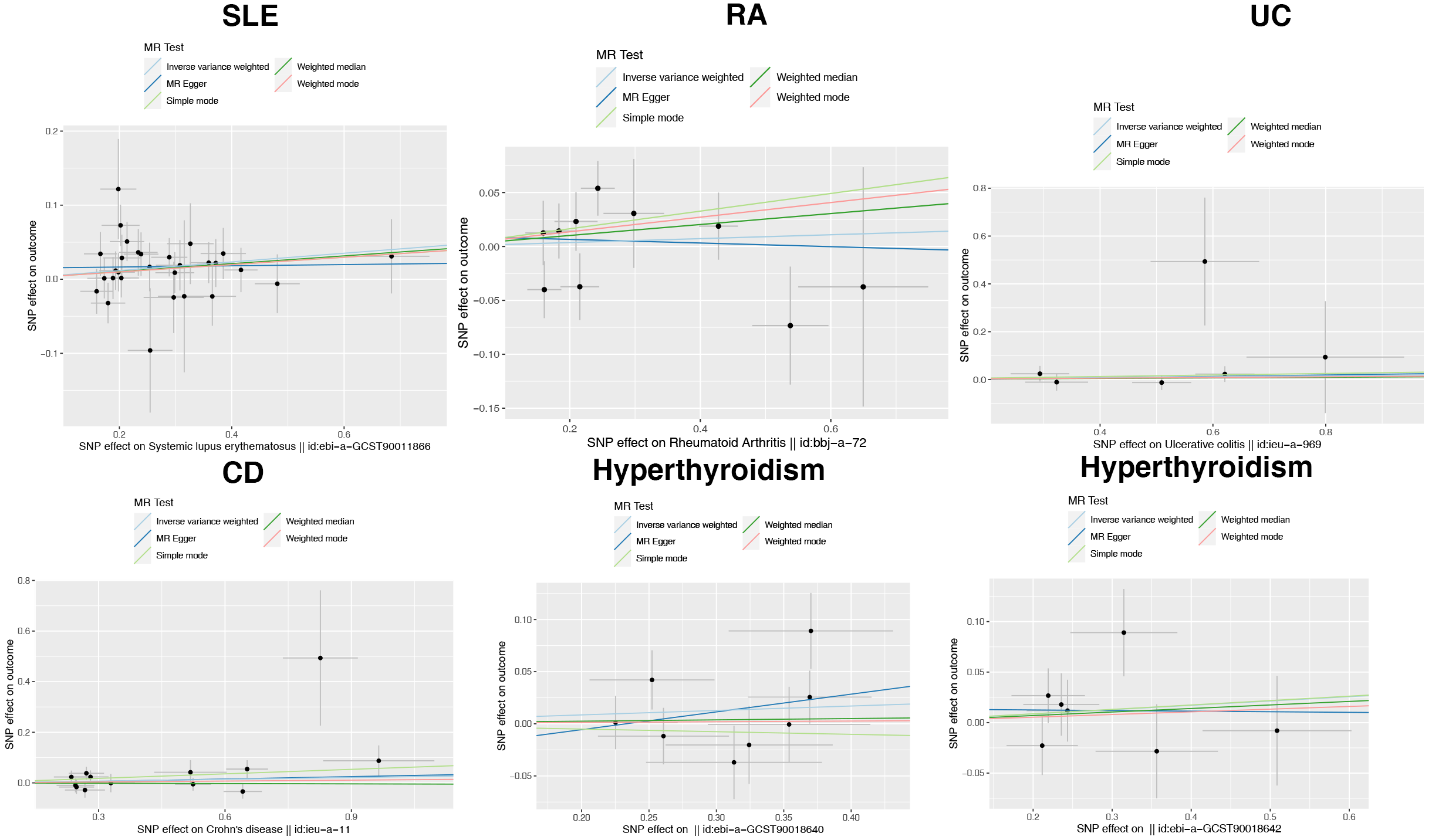


**Supplementary Figure 2b:Scatter plots of causal effect estimates for East Asian.**

2c


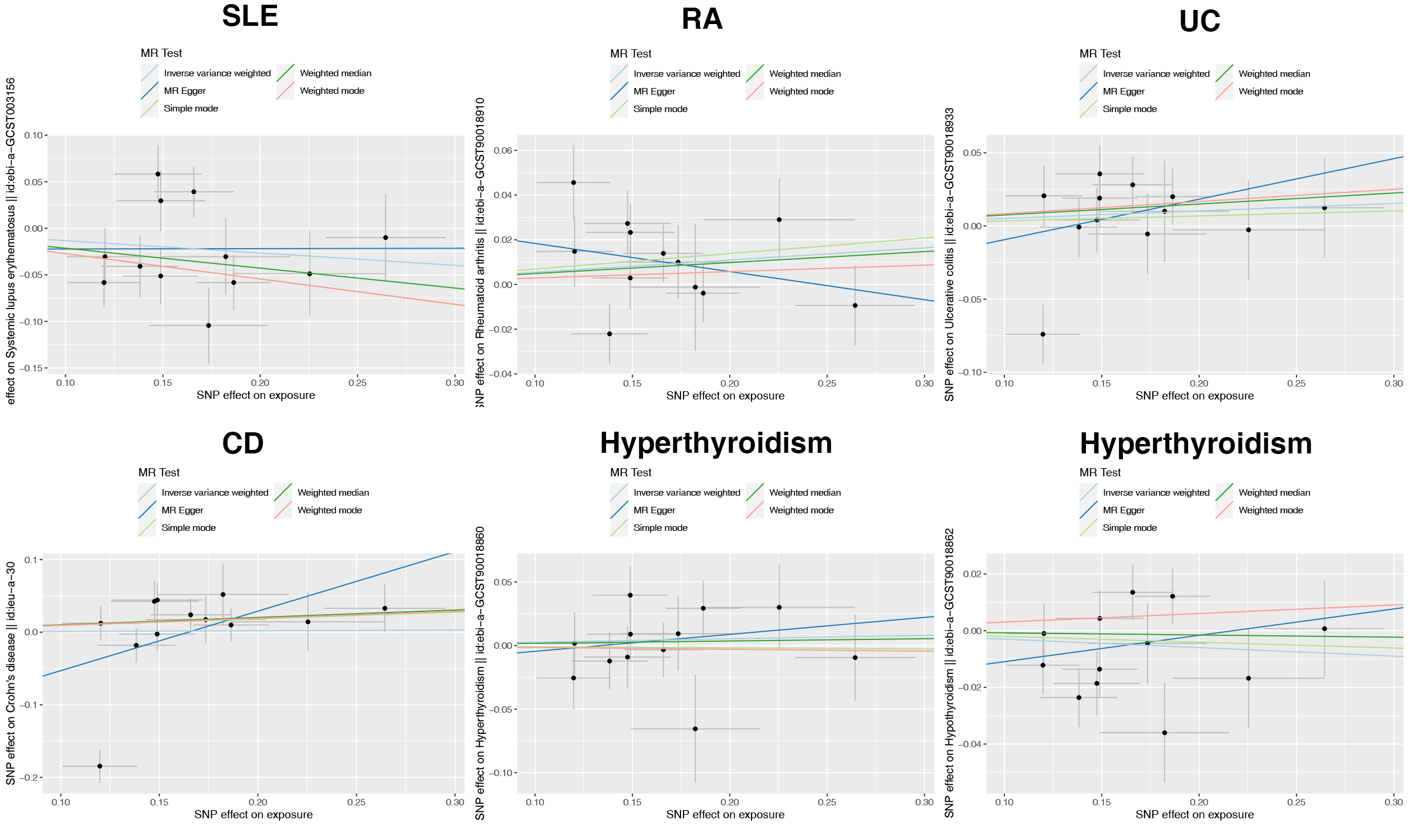


**Supplementary Figure 2c:Scatter plots of causal effect estimates for European in reverse MR.**

2d


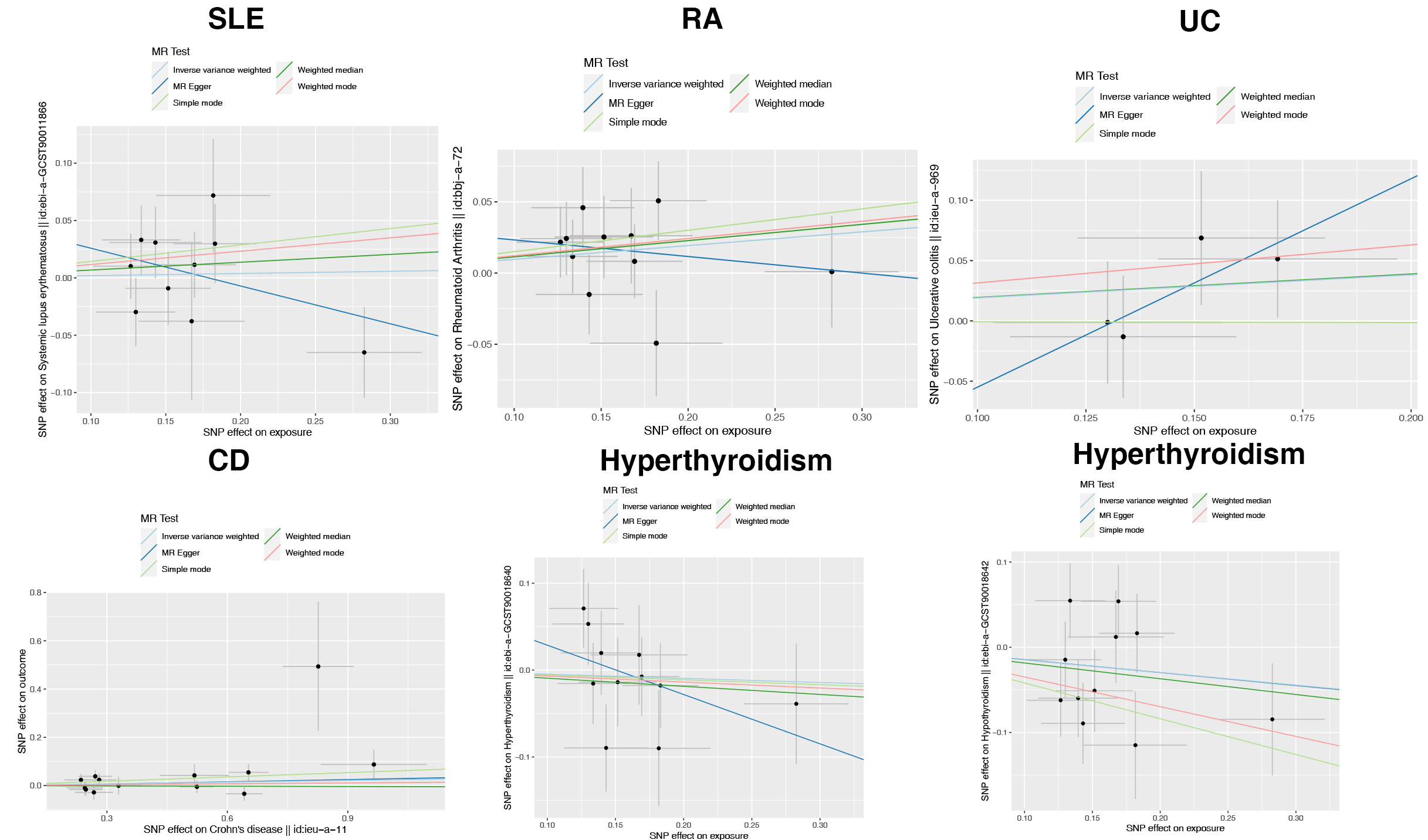


**Supplementary Figure 2d:Scatter plots of causal effect estimates for East Asian in reverse MR.**

SLE, Systemic lupus erythematosus; RA, Rheumatoid arthritis; UC, Ulcerative colitis; CD, Crohn's disease;

**3a**

**
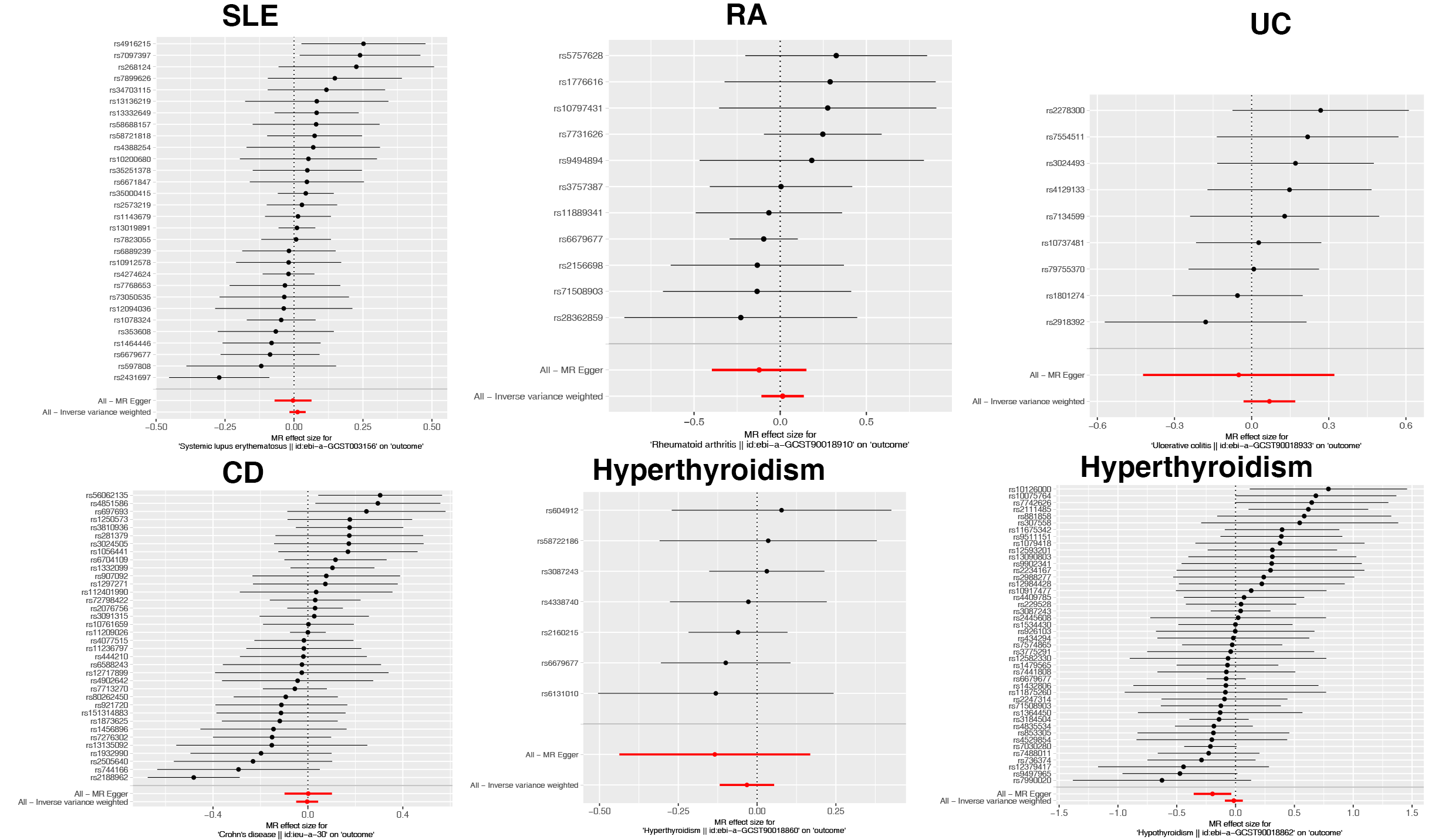
**

**Supplementary Figure 3a：Forest plot of causal effect estimates for European.**

**3b**

**
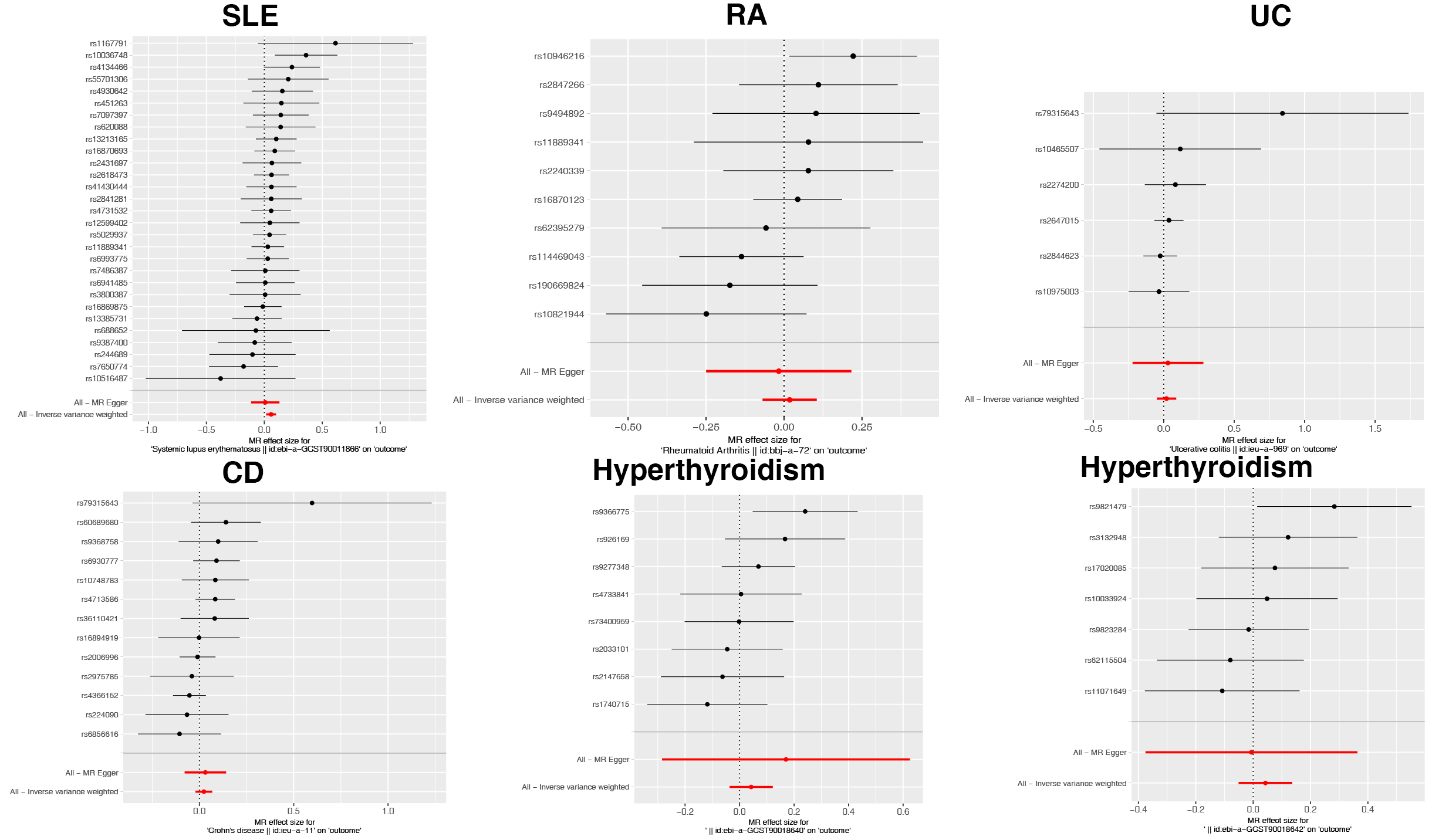
**

**Supplementary Figure 3b：Forest plot of causal effect estimates for East Asian.**

**3c**

**
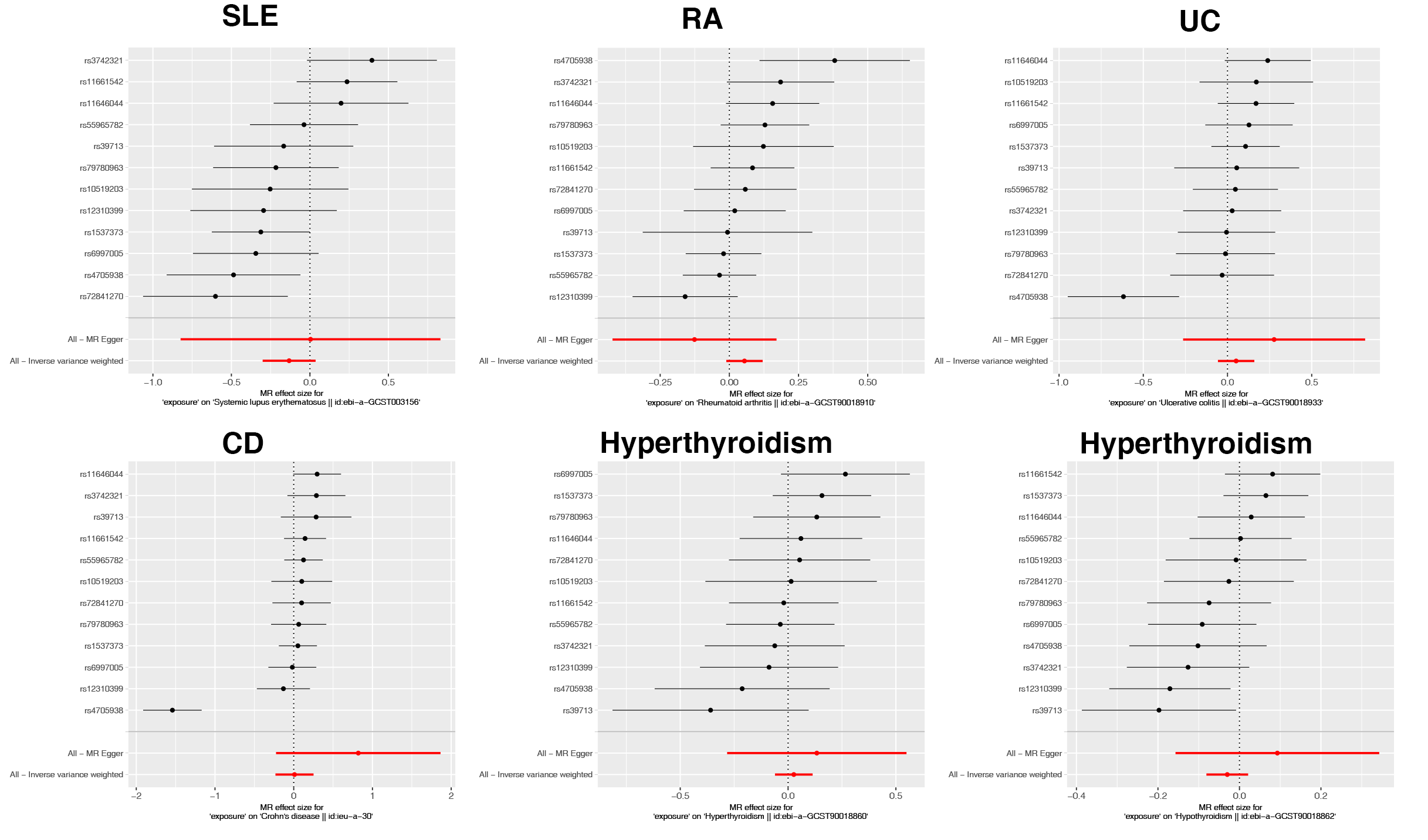
**

**Supplementary Figure 3c：Forest plot of causal effect estimates for European in revese.**

**3d**

**
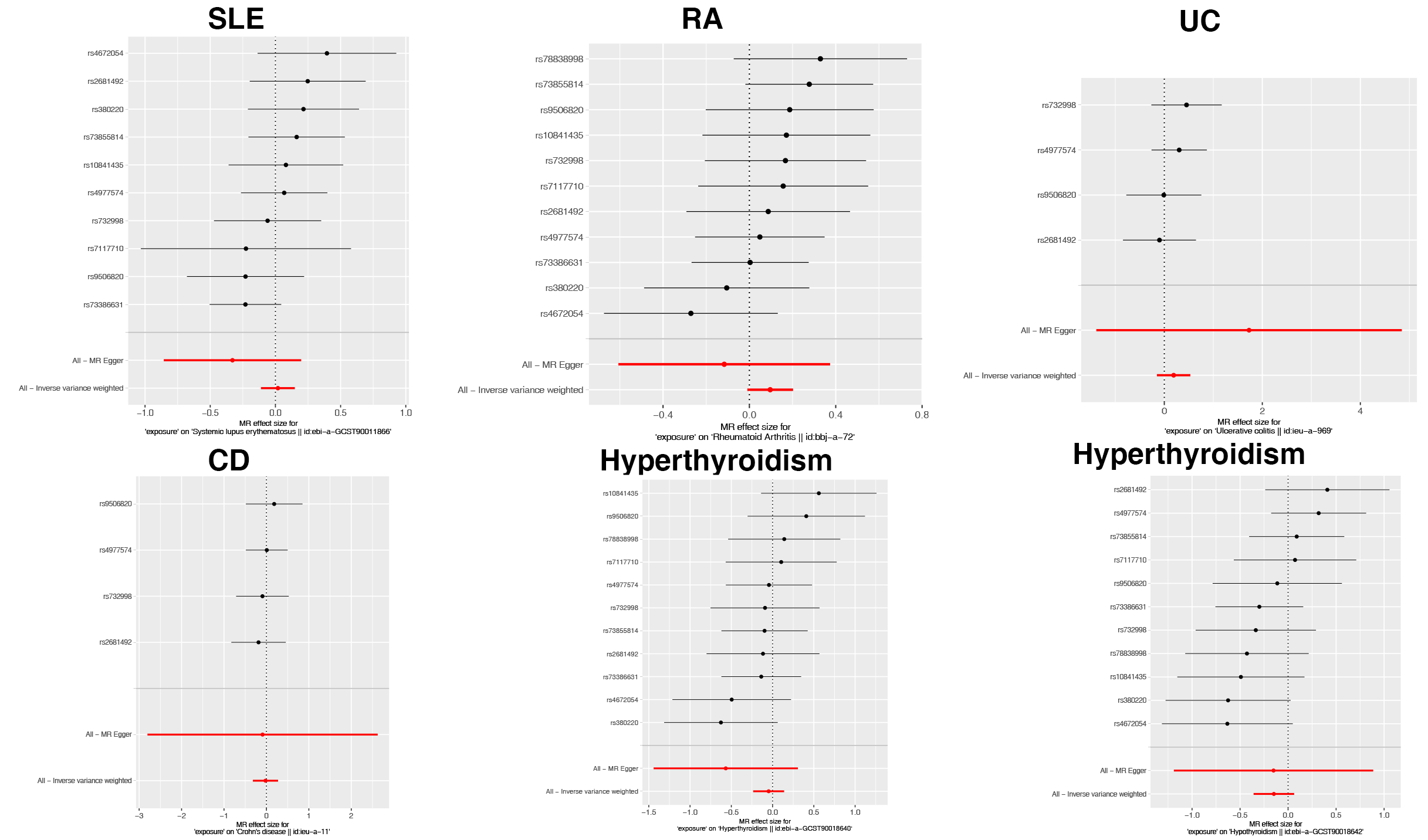
**

**Supplementary Figure 3c：Forest plot of causal effect estimates for East Asian in revese.**

SLE, Systemic lupus erythematosus; RA, Rheumatoid arthritis; UC, Ulcerative colitis; CD, Crohn's disease;


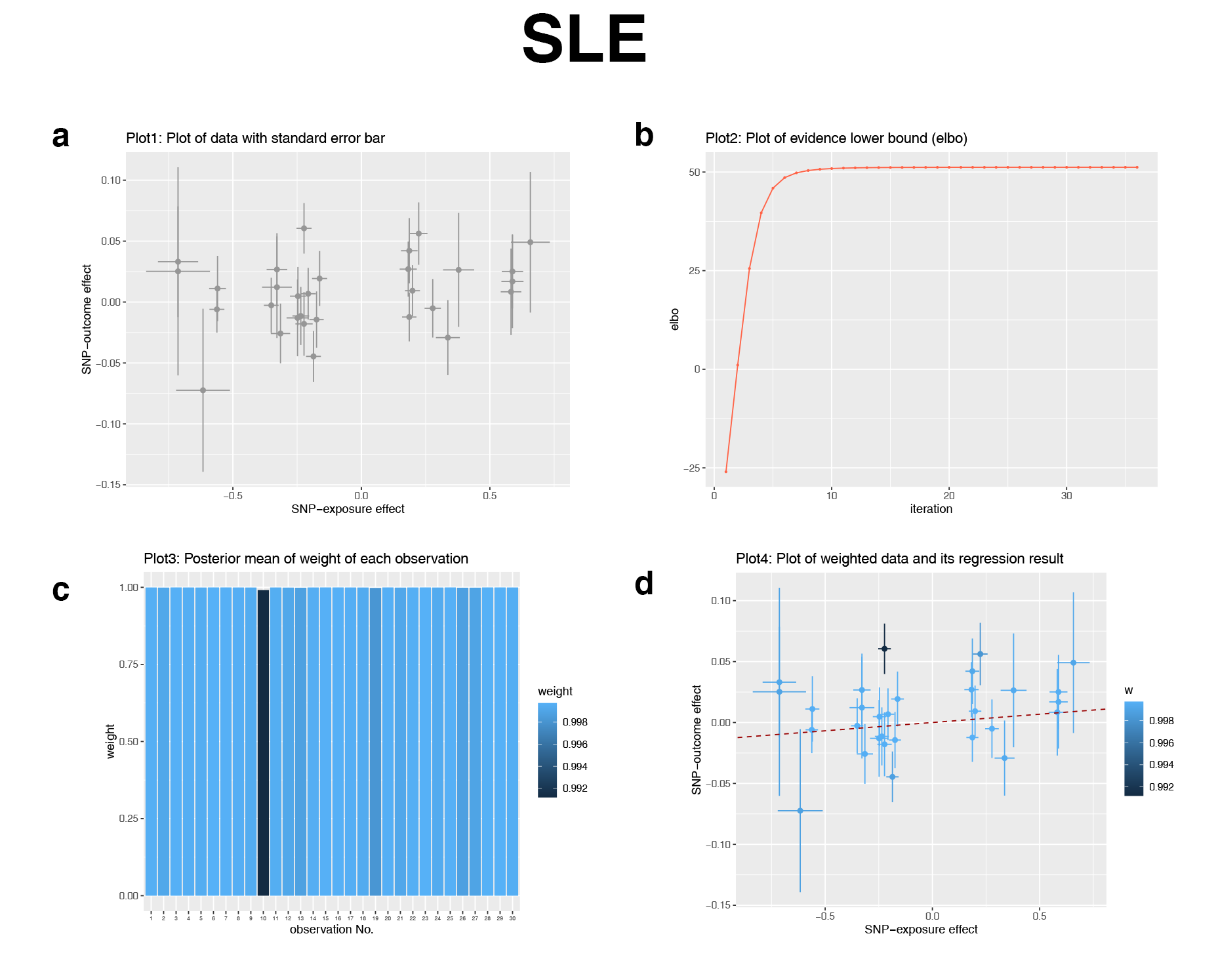


**Supplementary Figure 4:BWMR analyses of SLE with IA in European population.**

1. Plot of data with standard error bar in BWMR. Dots represent the estimated causal effect sizes (Beta), and their standard errors (SE) are represented by bars; b. Plot of evidence lower in BWMR; c. Posterior means of the weight of each observation in BWMR, valid SNPs were assigned close to 1 and outliers were adaptively down-weighted by BWMR; d. Plot of weighted data and its regression result in BWMR. The dots represent the causal effect (Beta) for each SNP; the bars represent their standard errors (SE); the dashed line indicates the regression slope for BWMR, and the depth of blue means the weight.


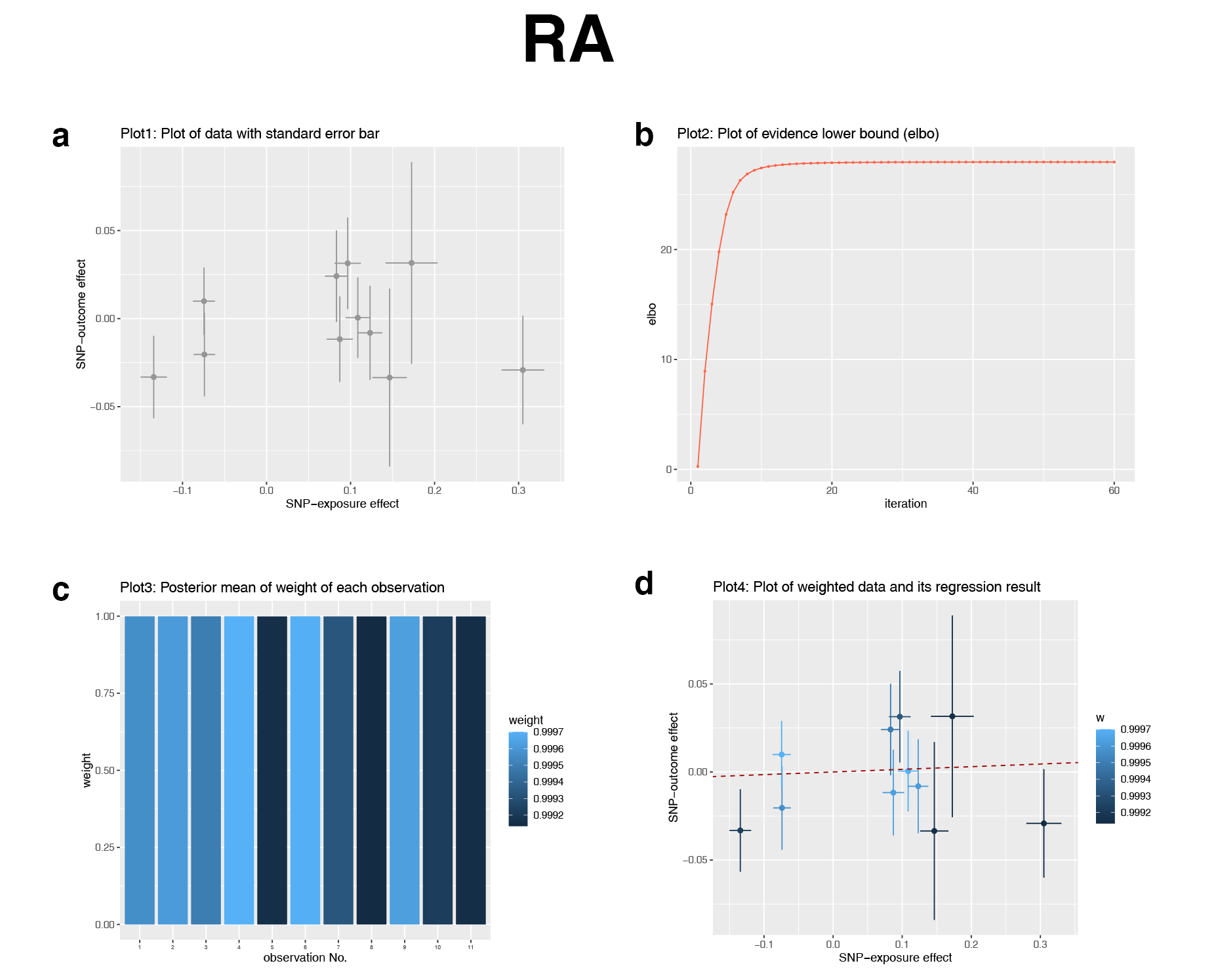


**Supplementary Figure 5:BWMR analyses of RA with IA in European population.**

1. Plot of data with standard error bar in BWMR. Dots represent the estimated causal effect sizes (Beta), and their standard errors (SE) are represented by bars; b. Plot of evidence lower in BWMR; c. Posterior means of the weight of each observation in BWMR, valid SNPs were assigned close to 1 and outliers were adaptively down-weighted by BWMR; d. Plot of weighted data and its regression result in BWMR. The dots represent the causal effect (Beta) for each SNP; the bars represent their standard errors (SE); the dashed line indicates the regression slope for BWMR, and the depth of blue means the weight.


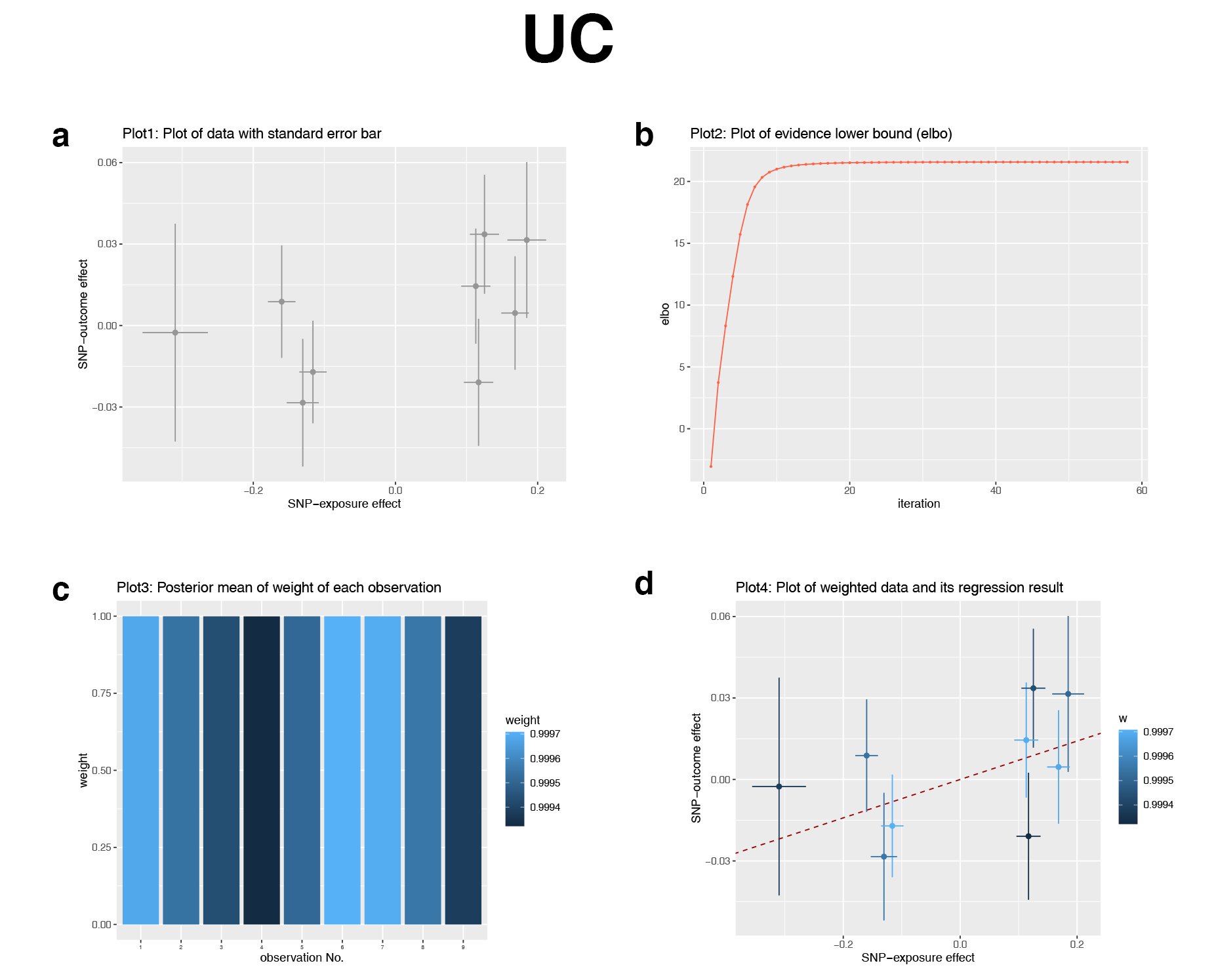


**Supplementary Figure 6:BWMR analyses of UC with IA in European population.**

1. Plot of data with standard error bar in BWMR. Dots represent the estimated causal effect sizes (Beta), and their standard errors (SE) are represented by bars; b. Plot of evidence lower in BWMR; c. Posterior means of the weight of each observation in BWMR, valid SNPs were assigned close to 1 and outliers were adaptively down-weighted by BWMR; d. Plot of weighted data and its regression result in BWMR. The dots represent the causal effect (Beta) for each SNP; the bars represent their standard errors (SE); the dashed line indicates the regression slope for BWMR, and the depth of blue means the weight.


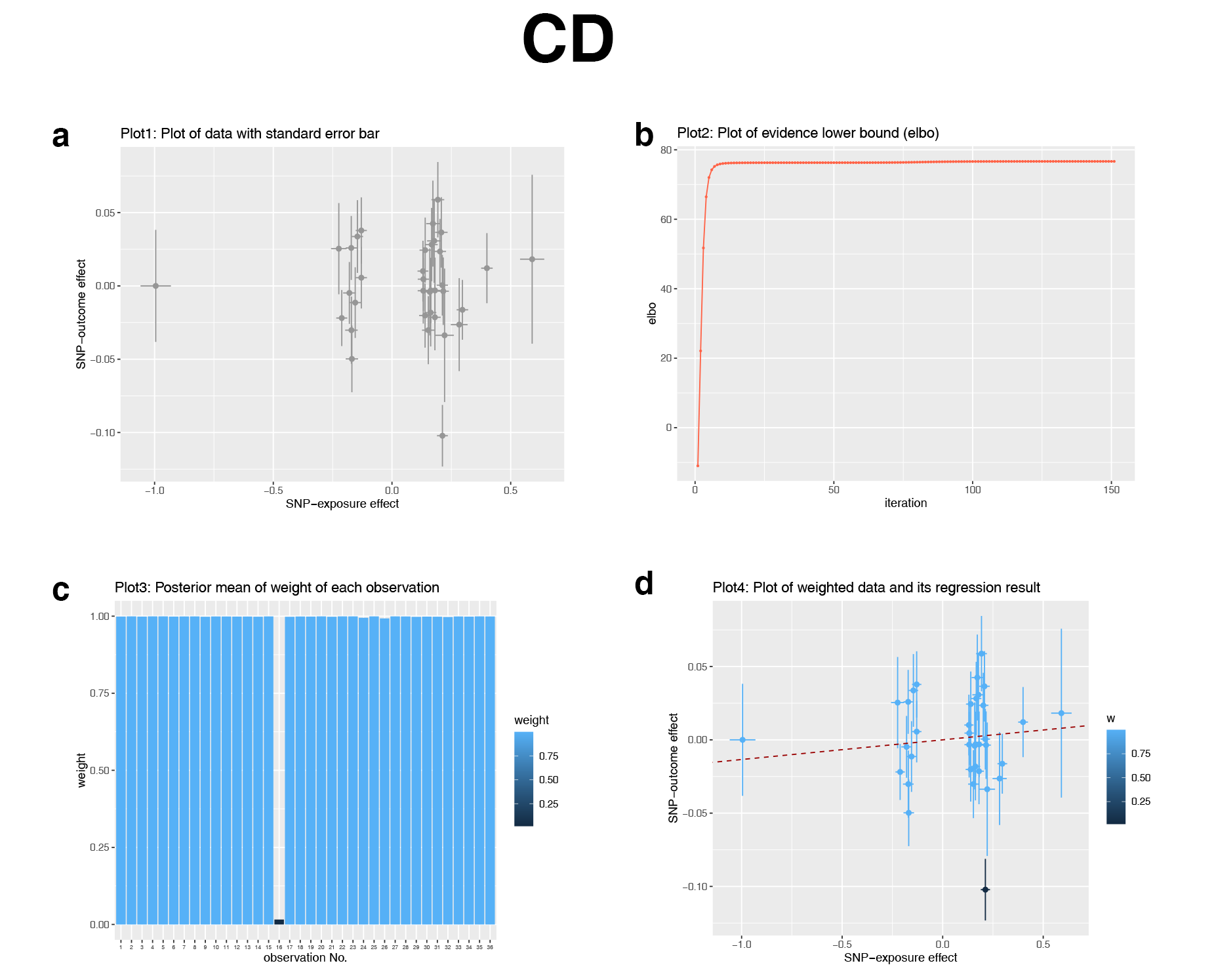


**Supplementary Figure 7:BWMR analyses of CD with IA in European population.**

1. Plot of data with standard error bar in BWMR. Dots represent the estimated causal effect sizes (Beta), and their standard errors (SE) are represented by bars; b. Plot of evidence lower in BWMR; c. Posterior means of the weight of each observation in BWMR, valid SNPs were assigned close to 1 and outliers were adaptively down-weighted by BWMR; d. Plot of weighted data and its regression result in BWMR. The dots represent the causal effect (Beta) for each SNP; the bars represent their standard errors (SE); the dashed line indicates the regression slope for BWMR, and the depth of blue means the weight.


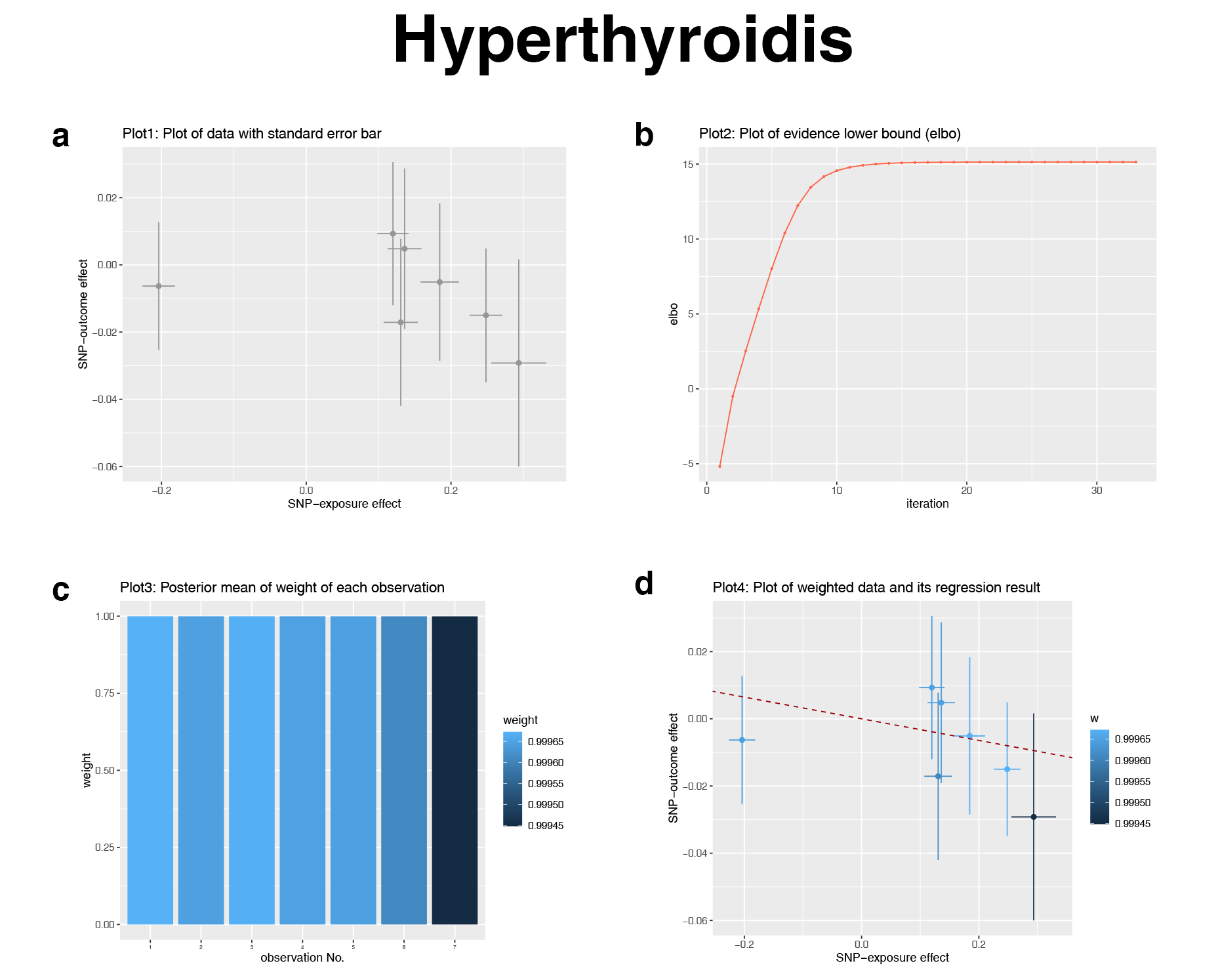


**Supplementary Figure 8:BWMR analyses of Hyperthyroidis with IA in European population.**

1. Plot of data with standard error bar in BWMR. Dots represent the estimated causal effect sizes (Beta), and their standard errors (SE) are represented by bars; b. Plot of evidence lower in BWMR; c. Posterior means of the weight of each observation in BWMR, valid SNPs were assigned close to 1 and outliers were adaptively down-weighted by BWMR; d. Plot of weighted data and its regression result in BWMR. The dots represent the causal effect (Beta) for each SNP; the bars represent their standard errors (SE); the dashed line indicates the regression slope for BWMR, and the depth of blue means the weight.


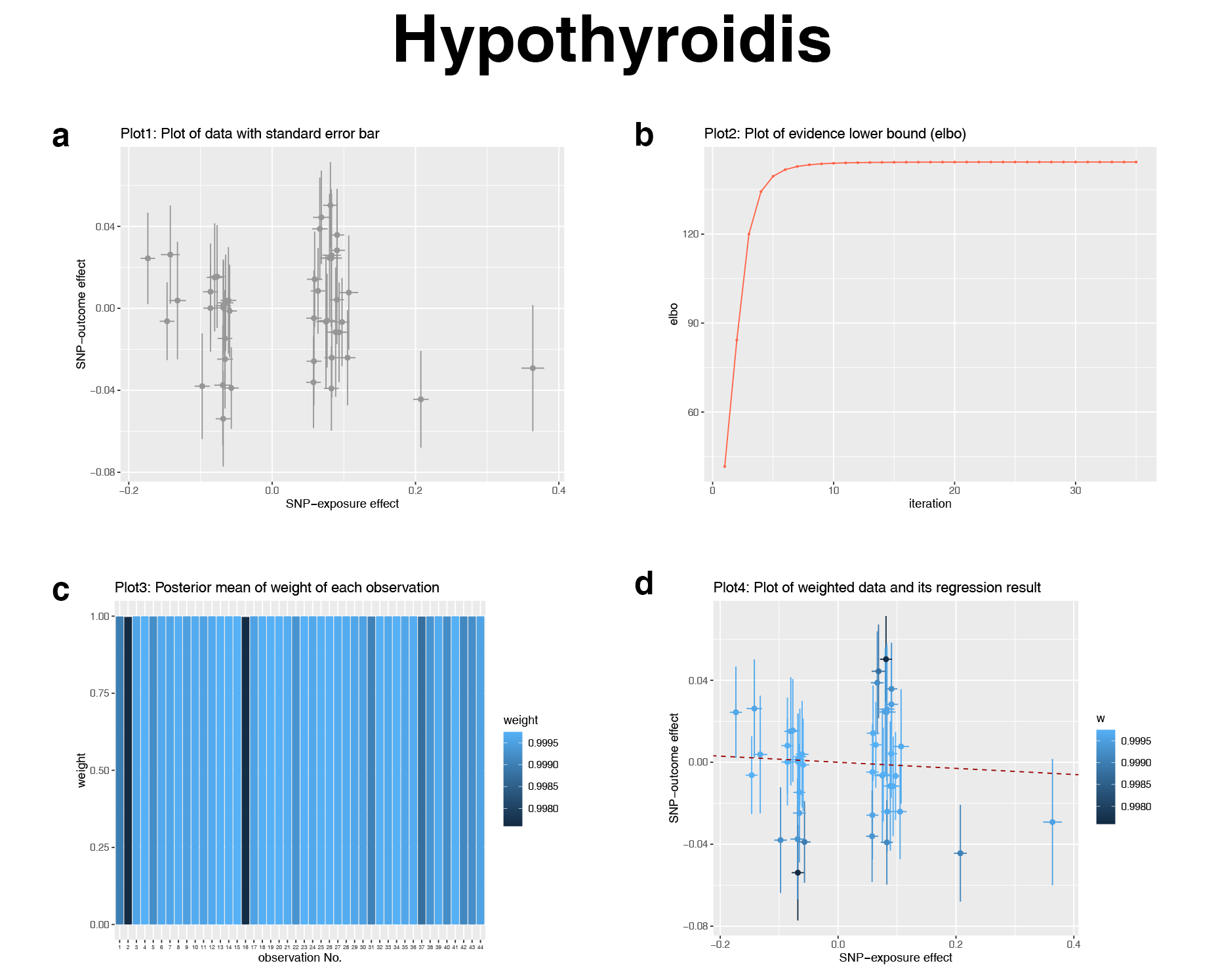


**Supplementary Figure 9:BWMR analyses of Hypothyroidis with IA in European population.**

1. Plot of data with standard error bar in BWMR. Dots represent the estimated causal effect sizes (Beta), and their standard errors (SE) are represented by bars; b. Plot of evidence lower in BWMR; c. Posterior means of the weight of each observation in BWMR, valid SNPs were assigned close to 1 and outliers were adaptively down-weighted by BWMR; d. Plot of weighted data and its regression result in BWMR. The dots represent the causal effect (Beta) for each SNP; the bars represent their standard errors (SE); the dashed line indicates the regression slope for BWMR, and the depth of blue means the weight.


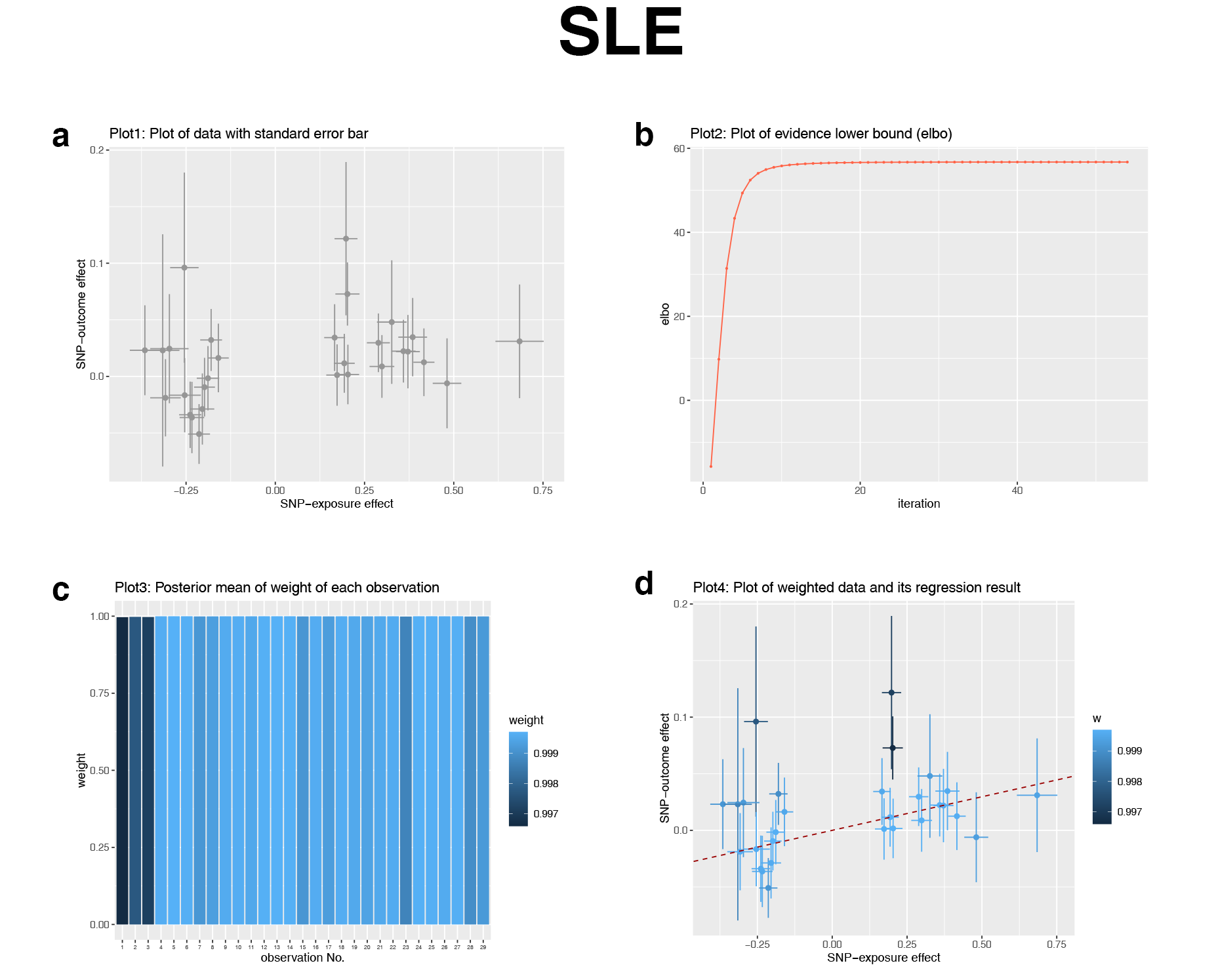


**Supplementary Figure 10:BWMR analyses of SLE with IA in East Asian population.**

1. Plot of data with standard error bar in BWMR. Dots represent the estimated causal effect sizes (Beta), and their standard errors (SE) are represented by bars; b. Plot of evidence lower in BWMR; c. Posterior means of the weight of each observation in BWMR, valid SNPs were assigned close to 1 and outliers were adaptively down-weighted by BWMR; d. Plot of weighted data and its regression result in BWMR. The dots represent the causal effect (Beta) for each SNP; the bars represent their standard errors (SE); the dashed line indicates the regression slope for BWMR, and the depth of blue means the weight.


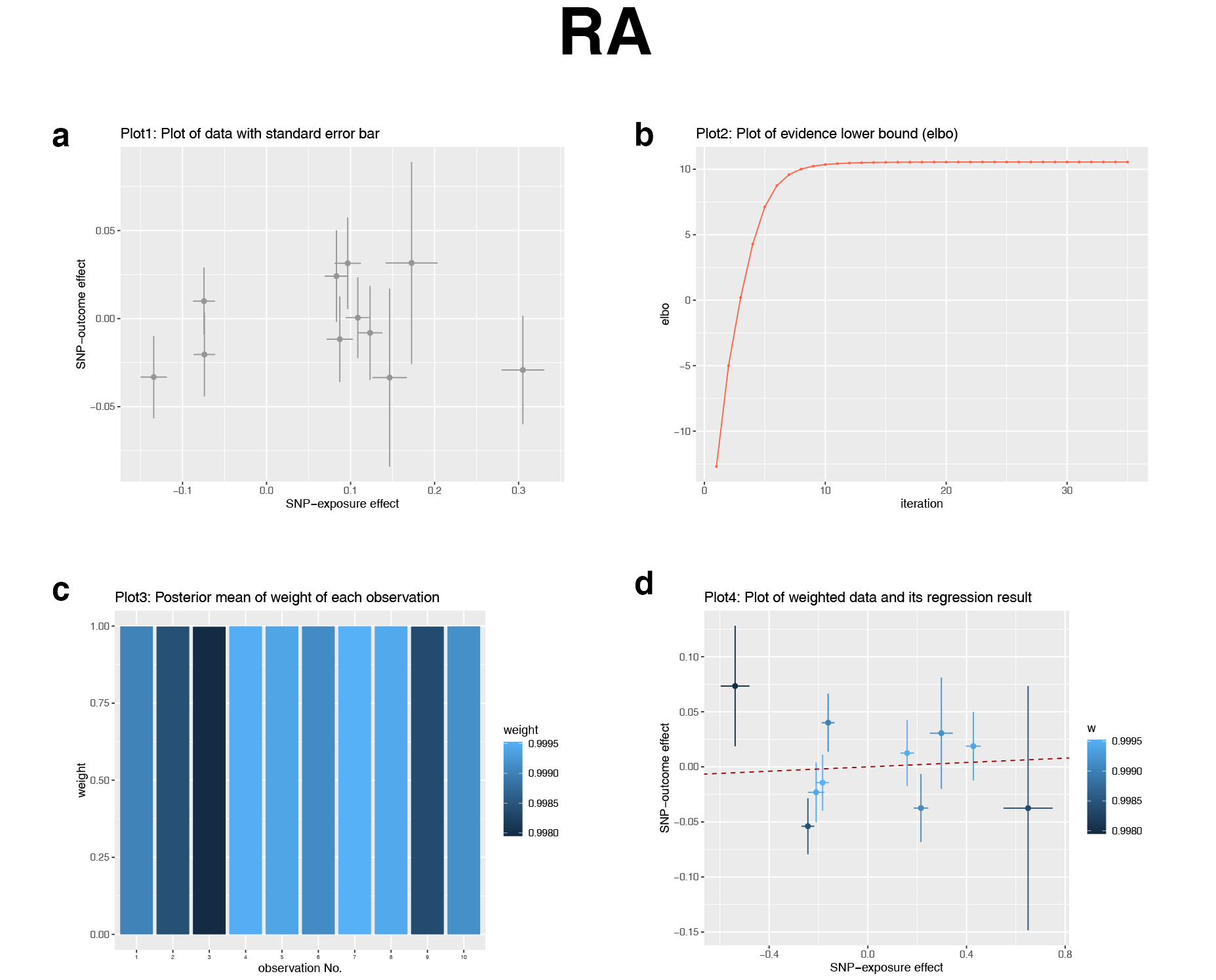


**Supplementary Figure 11:BWMR analyses of RA with IA in East Asian population.**

1. Plot of data with standard error bar in BWMR. Dots represent the estimated causal effect sizes (Beta), and their standard errors (SE) are represented by bars; b. Plot of evidence lower in BWMR; c. Posterior means of the weight of each observation in BWMR, valid SNPs were assigned close to 1 and outliers were adaptively down-weighted by BWMR; d. Plot of weighted data and its regression result in BWMR. The dots represent the causal effect (Beta) for each SNP; the bars represent their standard errors (SE); the dashed line indicates the regression slope for BWMR, and the depth of blue means the weight.


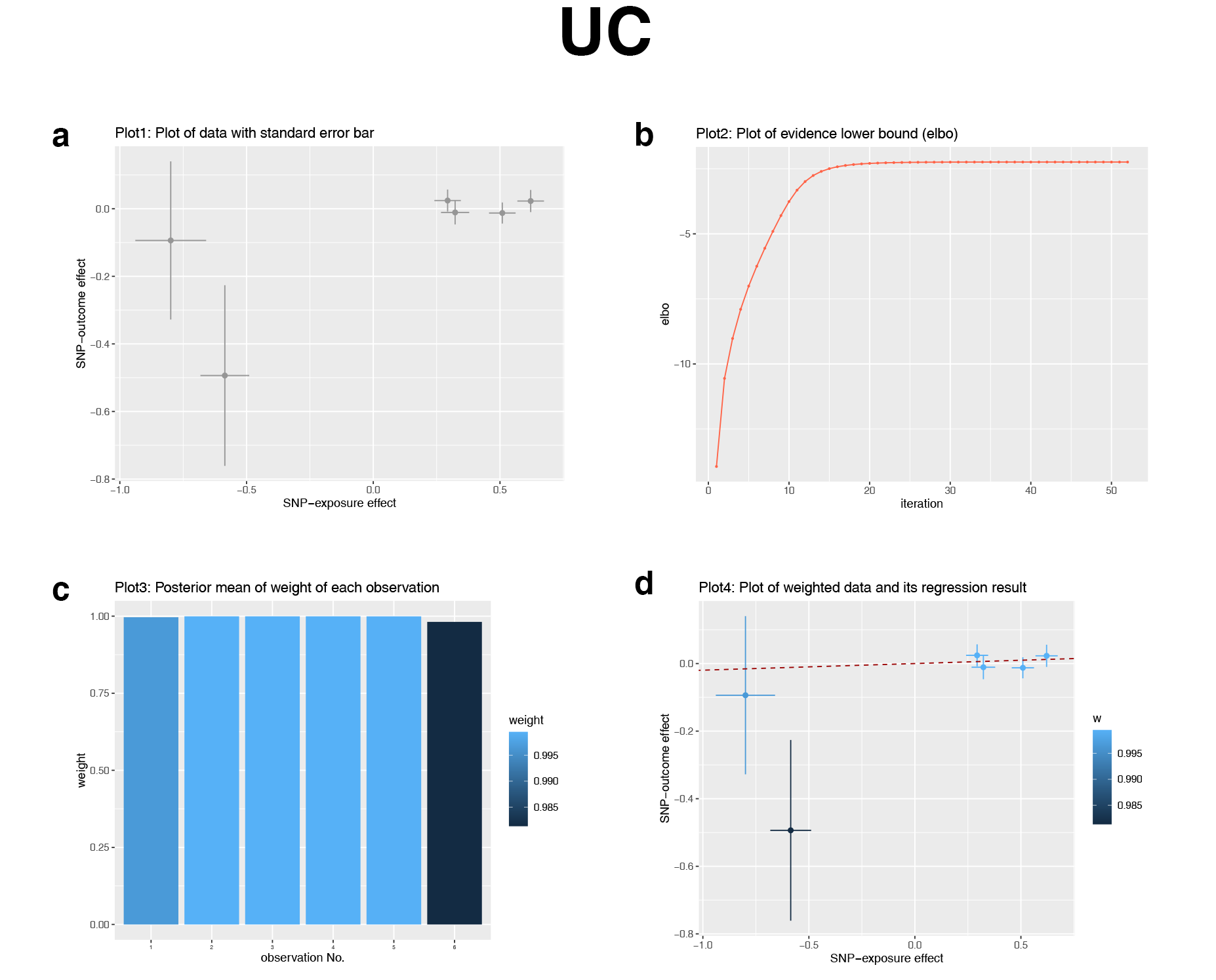


**Supplementary Figure 12:BWMR analyses of UC with IA in East Asian population.**

1. Plot of data with standard error bar in BWMR. Dots represent the estimated causal effect sizes (Beta), and their standard errors (SE) are represented by bars; b. Plot of evidence lower in BWMR; c. Posterior means of the weight of each observation in BWMR, valid SNPs were assigned close to 1 and outliers were adaptively down-weighted by BWMR; d. Plot of weighted data and its regression result in BWMR. The dots represent the causal effect (Beta) for each SNP; the bars represent their standard errors (SE); the dashed line indicates the regression slope for BWMR, and the depth of blue means the weight.


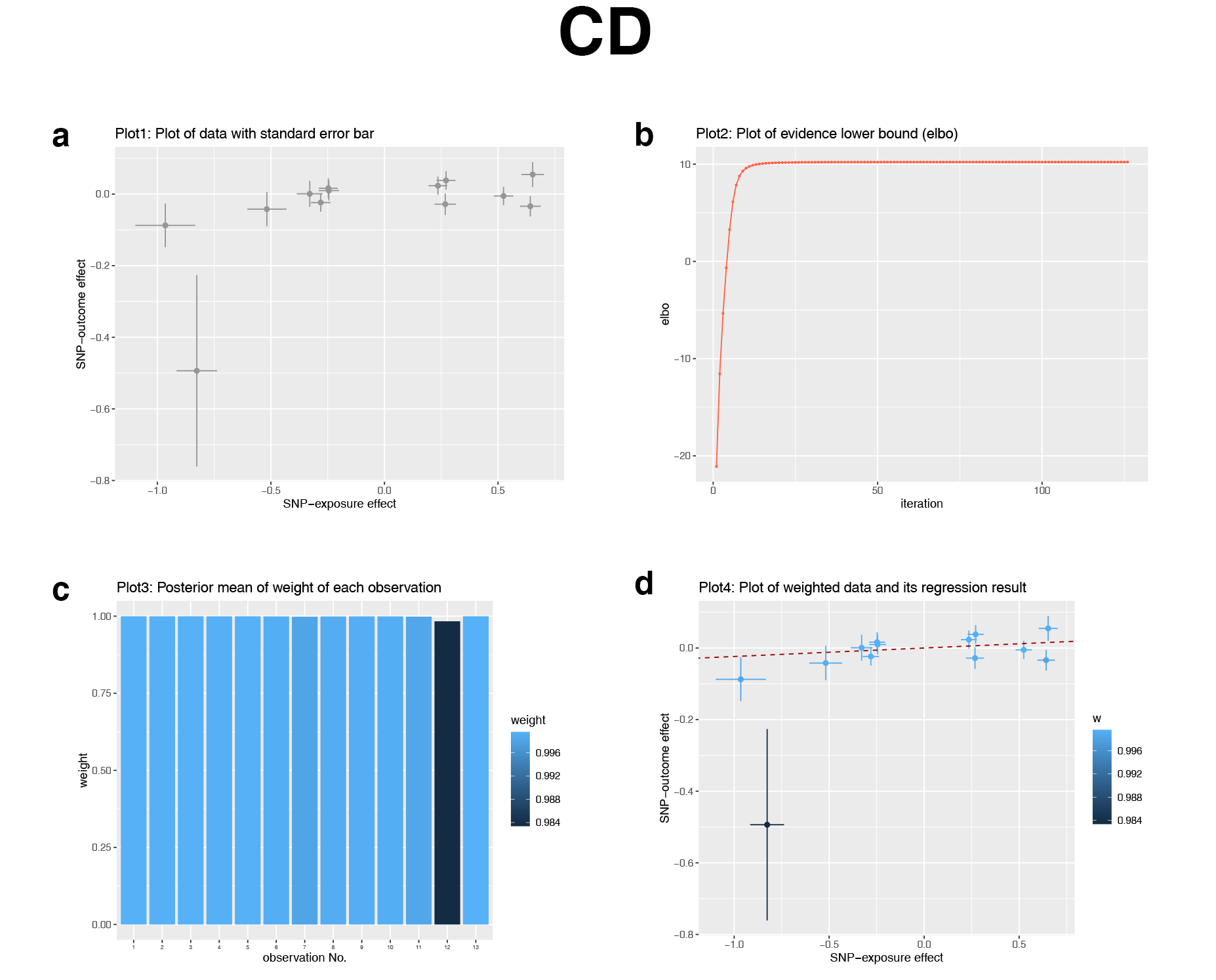


**Supplementary Figure 13:BWMR analyses of CD with IA in East Asian population.**

1. Plot of data with standard error bar in BWMR. Dots represent the estimated causal effect sizes (Beta), and their standard errors (SE) are represented by bars; b. Plot of evidence lower in BWMR; c. Posterior means of the weight of each observation in BWMR, valid SNPs were assigned close to 1 and outliers were adaptively down-weighted by BWMR; d. Plot of weighted data and its regression result in BWMR. The dots represent the causal effect (Beta) for each SNP; the bars represent their standard errors (SE); the dashed line indicates the regression slope for BWMR, and the depth of blue means the weight.


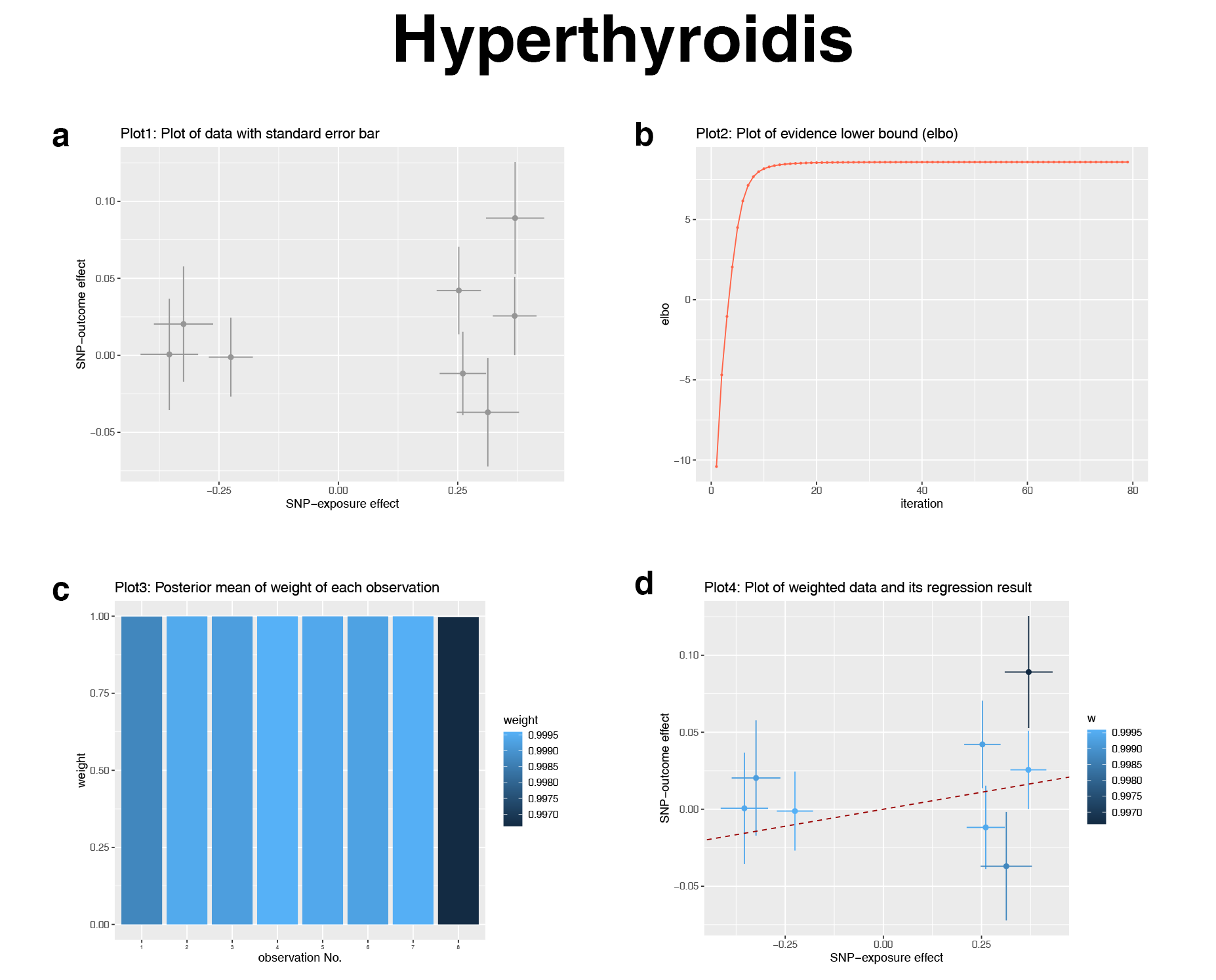


**Supplementary Figure 14:BWMR analyses of Hypothyroidis with IA in East Asian population.**

1. Plot of data with standard error bar in BWMR. Dots represent the estimated causal effect sizes (Beta), and their standard errors (SE) are represented by bars; b. Plot of evidence lower in BWMR; c. Posterior means of the weight of each observation in BWMR, valid SNPs were assigned close to 1 and outliers were adaptively down-weighted by BWMR; d. Plot of weighted data and its regression result in BWMR. The dots represent the causal effect (Beta) for each SNP; the bars represent their standard errors (SE); the dashed line indicates the regression slope for BWMR, and the depth of blue means the weight.


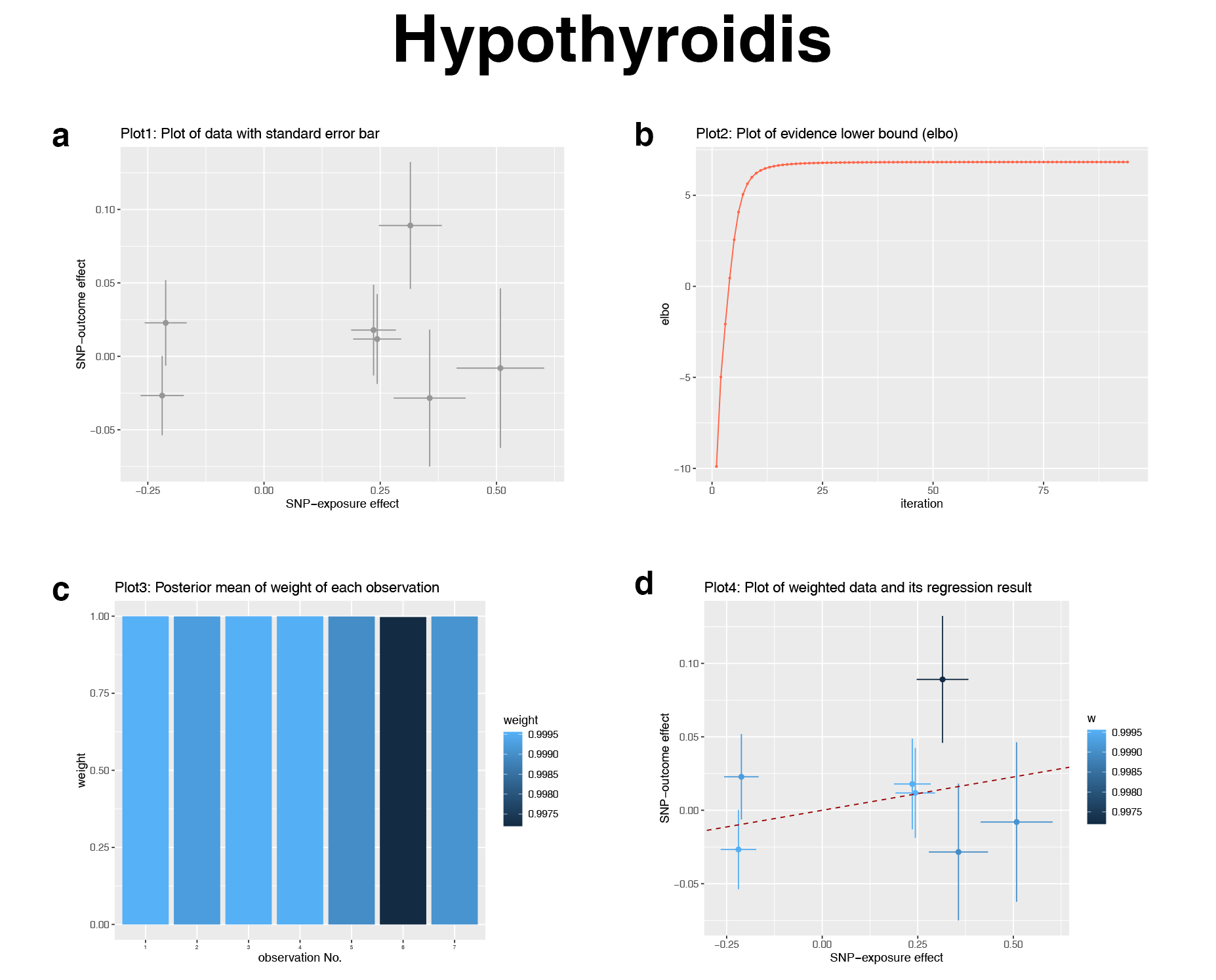


**Supplementary Figure 15:BWMR analyses of Hypothyroidis with IA in East Asian population.**

1. Plot of data with standard error bar in BWMR. Dots represent the estimated causal effect sizes (Beta), and their standard errors (SE) are represented by bars; b. Plot of evidence lower in BWMR; c. Posterior means of the weight of each observation in BWMR, valid SNPs were assigned close to 1 and outliers were adaptively down-weighted by BWMR; d. Plot of weighted data and its regression result in BWMR. The dots represent the causal effect (Beta) for each SNP; the bars represent their standard errors (SE); the dashed line indicates the regression slope for BWMR, and the depth of blue means the weight.

a
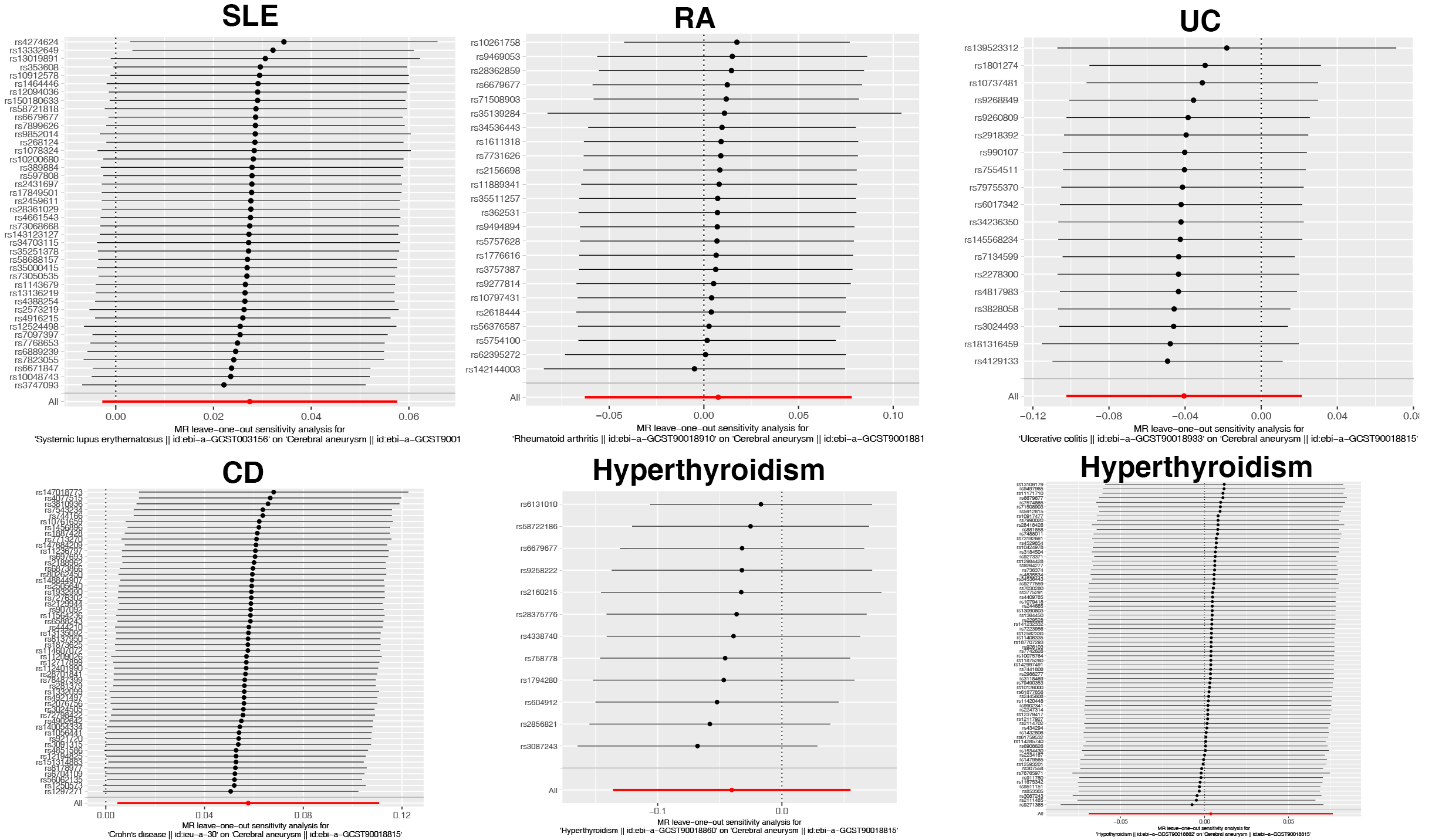


**Supplementary Figure 16a：Leave-one-out test plot of causal effect estimates for European in validation group.**

b
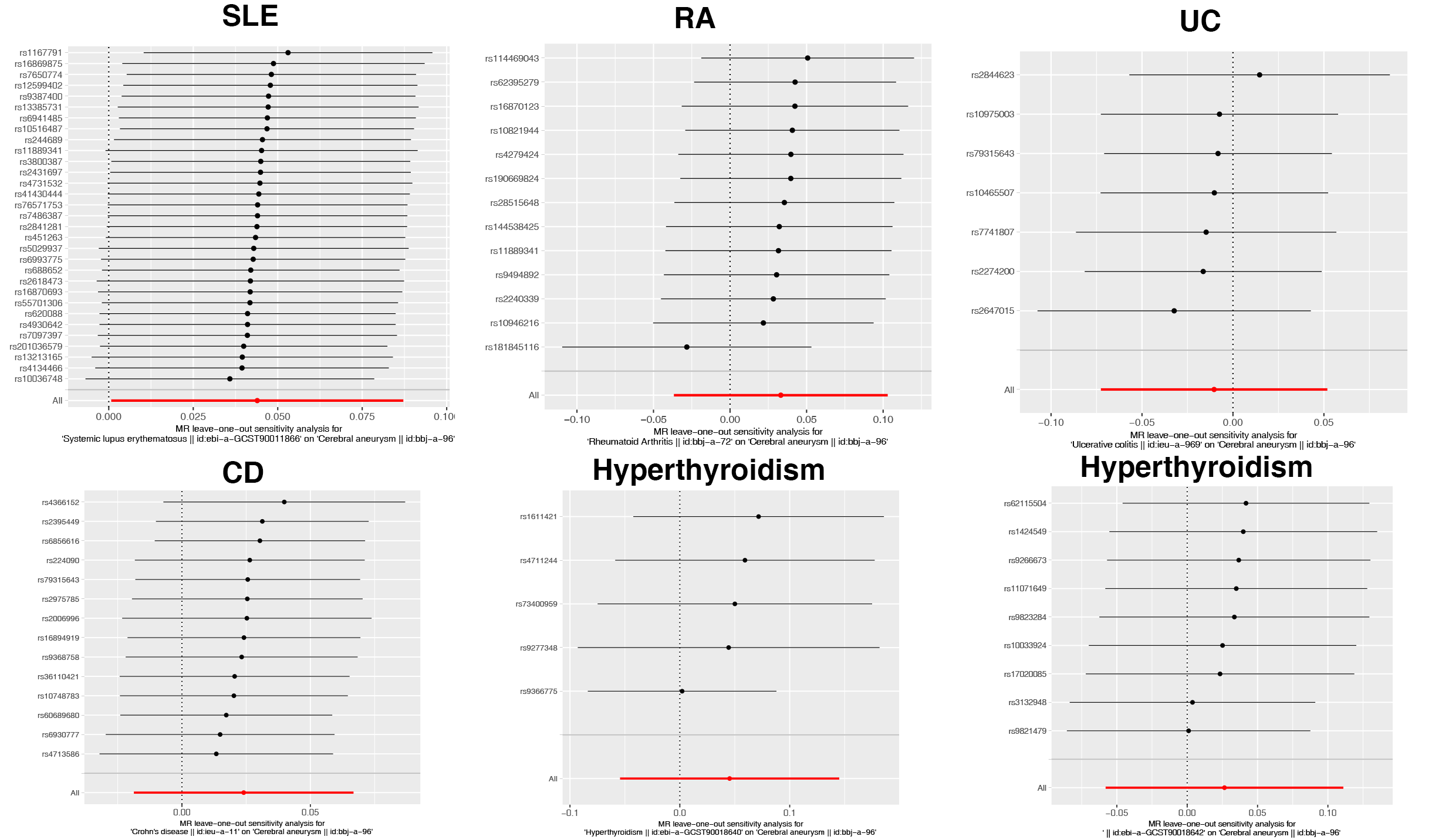


**Supplementary Figure 16b：Leave-one-out test plot of causal effect estimates for East Asian in validation group.**

a
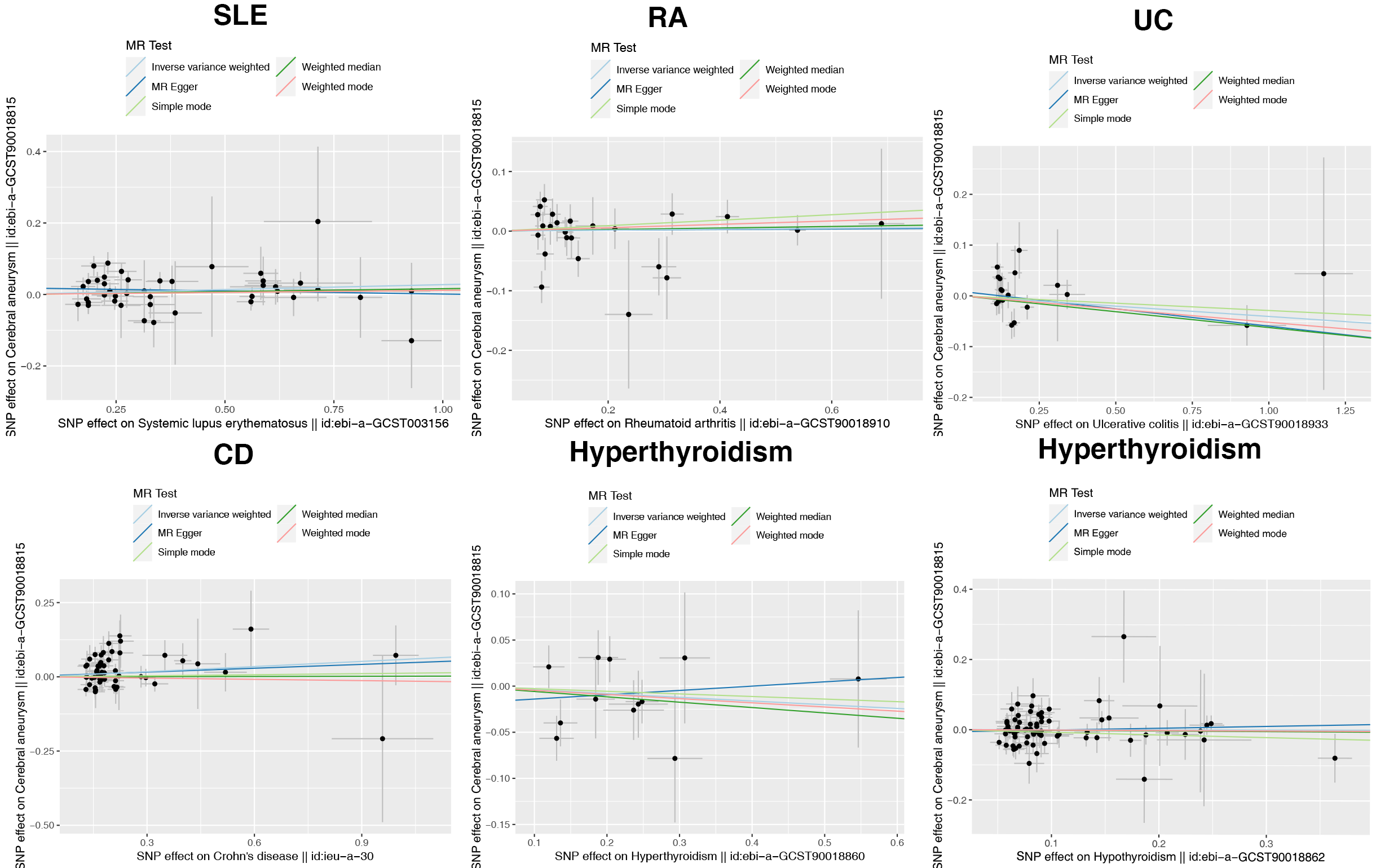


**Supplementary Figure 17a:Scatter plots of causal effect estimates for European in validation group.**

b


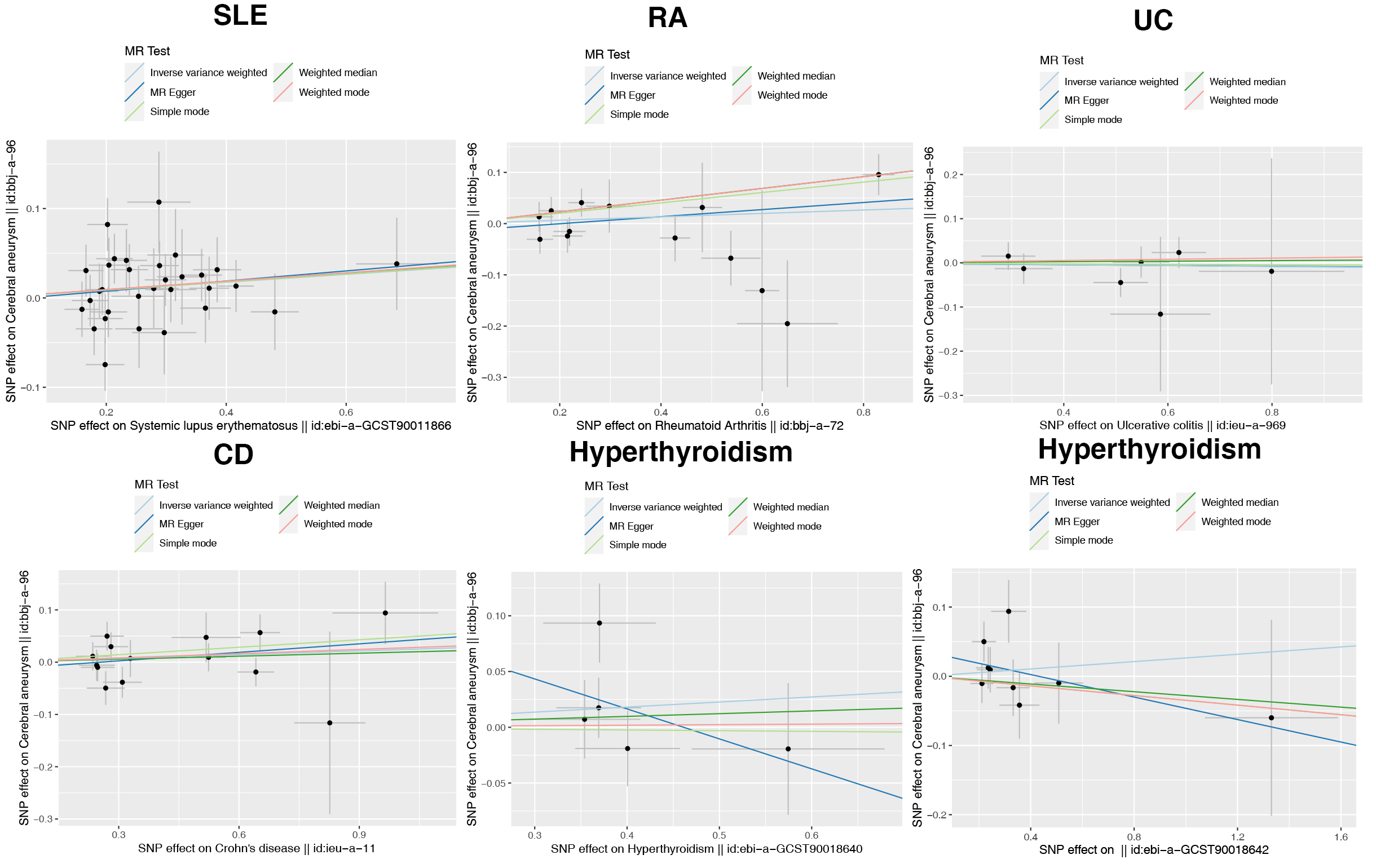


**Supplementary Figure 17b:Scatter plots of causal effect estimates for East Asian in validation group.**

a
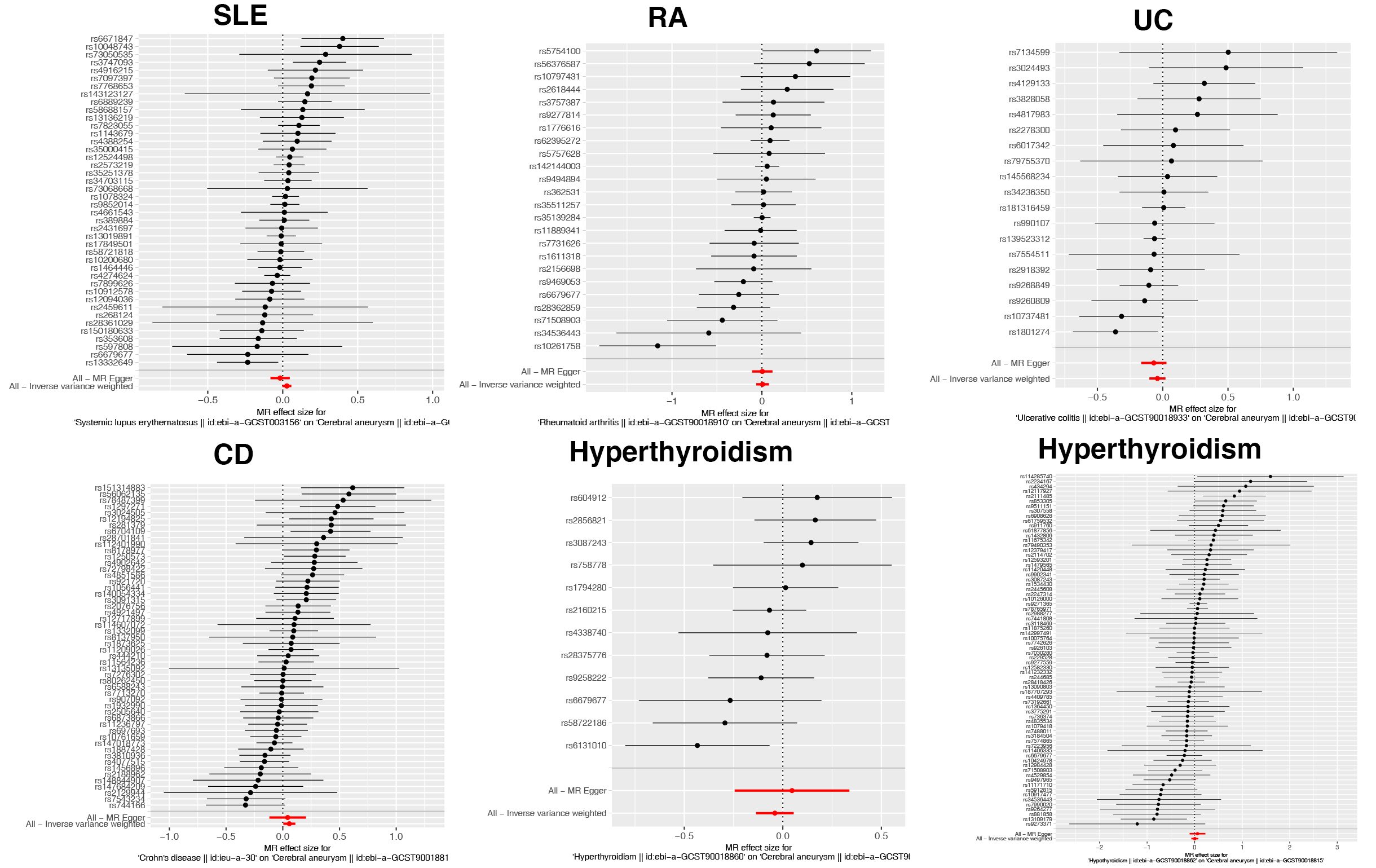


**Supplementary Figure 18a：Forest plot of causal effect estimates for European in validation group.**

**b**


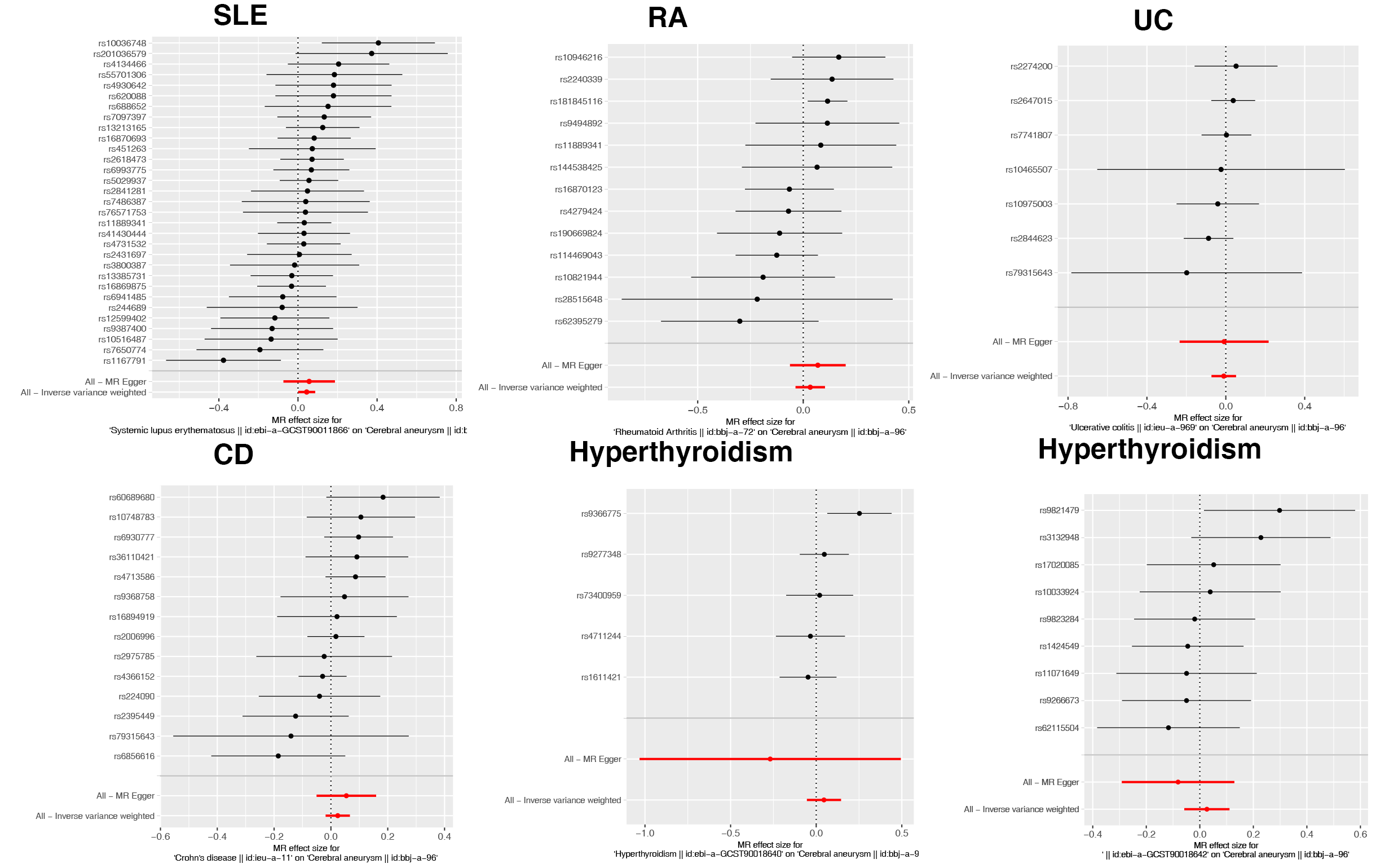


**Supplementary Figure 18b：Forest plot of causal effect estimates for East Asian in validation group.**
